# Inhibitory Effects of Novel 7-Substituted 6-iodo-3-*O*-Flavonol Glycosides against Cholinesterases and β-secretase Activities, and Evaluation for Potential Antioxidant Properties

**DOI:** 10.3390/molecules24193500

**Published:** 2019-09-26

**Authors:** Emmanuel N. Agbo, Samantha Gildenhuys, Malose J. Mphahlele

**Affiliations:** 1 Department of Chemistry, College of Science, Engineering and Technology, University of South Africa, Private Bag X06, Florida 1710, South Africa; piruesbest@yahoo.com; 2 Department of Life & Consumer Sciences, College of Agriculture and Environmental Sciences, University of South Africa, Private Bag X06, Florida 1710, South Africa

**Keywords:** 3-*O*-flavonol glycosides, X-ray, acetylcholinesterase, butyrylcholinesterase, β-secretase, kinetic studies, molecular docking, antioxidant assay

## Abstract

A series of 7-halogeno- (X = F, Cl, Br) and 7-methoxy-substituted acetylated 6-iodo-3-*O*-flavonol glycosides were prepared, and evaluated for inhibitory effect in vitro against acetylcholinesterase (AChE) and butyrylcholinesterase (BChE) activities. 7-Bromo-2-(4-chlorophenyl)-6-iodo-4*H*-chromen-4-one-3-*O*-2,3,4,6*-O*-tetraacetyl-β-*d*-glucopyranoside (**2k**) and 7-bromo-6-iodo-2-(4-methoxyphenyl)-4*H*-chromen-4-one-3-*O*-2,3,4,6-*O*-tetraacetyl-β-*d*-glucopyranoside (**2l**) exhibited significant inhibitory effect against AChE activity when compared to the activity of the reference standard, donepezil. Compound **2k** was found to be selective against AChE and to exhibit reduced inhibitory effect against BChE activity. 6-Iodo-7-methoxy-2-(4-methoxyphenyl)-4*H*-chromen-4-one-3-*O*-2,3,4,6-*O*-tetraacetyl-β-*d*-glucopyranoside (**2p**) was found to exhibit increased activity against BChE, more so than the activity of donepezil. The most active compounds were also evaluated for inhibitory effect against β-secretase activity and for potential radical scavenging activities. The experimental data were complemented with molecular docking (in silico) studies of the most active compounds into the active sites of these enzymes.

## 1. Introduction

Alzheimer’s disease (AD) is a chronic neurodegenerative disease, which is characterized by progressive loss of memory, cognitive impairment or inability to learn, and abnormal behavior [1]. The disease affects elderly people throughout the world and it usually starts slowly and worsens over time. It has also been found that the efficiency of the brain’s antioxidant system gradually decreases with age, and this is more so in AD patients [2]. Several molecular mechanisms of AD have been proposed, including the cholinergic hypothesis, β-amyloid cascade, oxidative stress, and metal imbalance [3]. Strategies currently pursued for the treatment of AD include inhibition of cholinesterases and targeting amyloid-β (Aβ) peptides as well as metal-Aβ complexes [4]. However, the pathogenesis of AD is far more complex with other mechanisms, with inflammation [5] and immune suppression [6] also implicated. The brains of patients suffering from AD, on the other hand, are characterized by significant oxidative damage associated with the abnormal marked accumulation of Aβ and the deposition of neurofibrillary tangles [7]. This has led to a growing debate for the inclusion of antioxidants as supplements in the standard therapy for AD [8]. However, a single multi-target drug may have distinctive advantages over drug-combination therapies for the treatment of a multi-factorial disease such as AD. This multi-target-directed ligand (MTDL) design strategy has been found to be more effective for the treatment of AD than the one-target-one drug concept and drug-combination therapies [9,10,11]. Moreover, MTDLs reduce the risk of poor patient compliance, drug–drug interactions, and pharmacokinetic differences between the individual drugs. It is evident that the treatment of AD could benefit from the use of multipotent drugs that present cholinesterases and β-secretase inhibitory activities as well as free-radical scavenging properties.

Plant-derived flavonoid-rich foods or supplements are envisaged to delay the initiation or slow the progression of AD and related neurodegenerative disorders [12]. Certain flavonols and flavones have been reported to act as β-secretase inhibitors and to suppress β-secretase expression [13]. Moreover, these flavonoids have been proven to be powerful antioxidants [14]. Flavonols exist predominantly in nature as the flavonol-3-*O*-glycosides. The sugar moiety affects the function, structure, and stability of cells and enzymes [15]. Glycosylation of quercetin to generate quercetin-3*-O*-glucoside, for example, has been found to prevent degradation of the flavonol scaffold and to increase biological activity [16]. The presence of halogen atom/s in a molecule has been found to increase the membrane permeability and prolongs the lifetime of the drug [17]. Moreover, the strong electron-withdrawing effect of the halogen atoms has been found to facilitate formation of hydrogen and/or halogen bonds that help to stabilize interactions of drug molecules with the protein targets [18,19,20]. A series of acetylated 3-*O*-flavonol glycosides was prepared before and their structure activity relationship for anticholinesterase effect evaluated with respect to the nature and pattern of the substituent on the B-ring [21]. Since no halogenated flavonoid glycosides exist in nature and are inspired by the MTDL strategy, a sugar moiety was appended onto the known 7-halogeno- (X = F, Cl, Br) and 7-methoxy-substituted 6-iodoflavonols [22]. The prepared halogenated 3-*O*-flavonol glycosides were evaluated through enzymatic assays in vitro for their potential to inhibit acetylcholinesterase (AChE) and butyrylcholinesterase (BChE) activities. Moreover, the most active compounds against these enzymes were evaluated for inhibitory potential against β-secretase and for antioxidant activity in the 2,2-diphenyl-1-picrylhydrazyl (DPPH) radical scavenging activity assay. Molecular docking (in silico) studies into the active sites of AChE, BChE, and β-secretase were performed on the most active compounds against these enzymes to rationalize the hypothetical protein–ligand binding interactions.

## 2. Results and Discussion

### 2.1. Chemistry

**Scheme 1:**
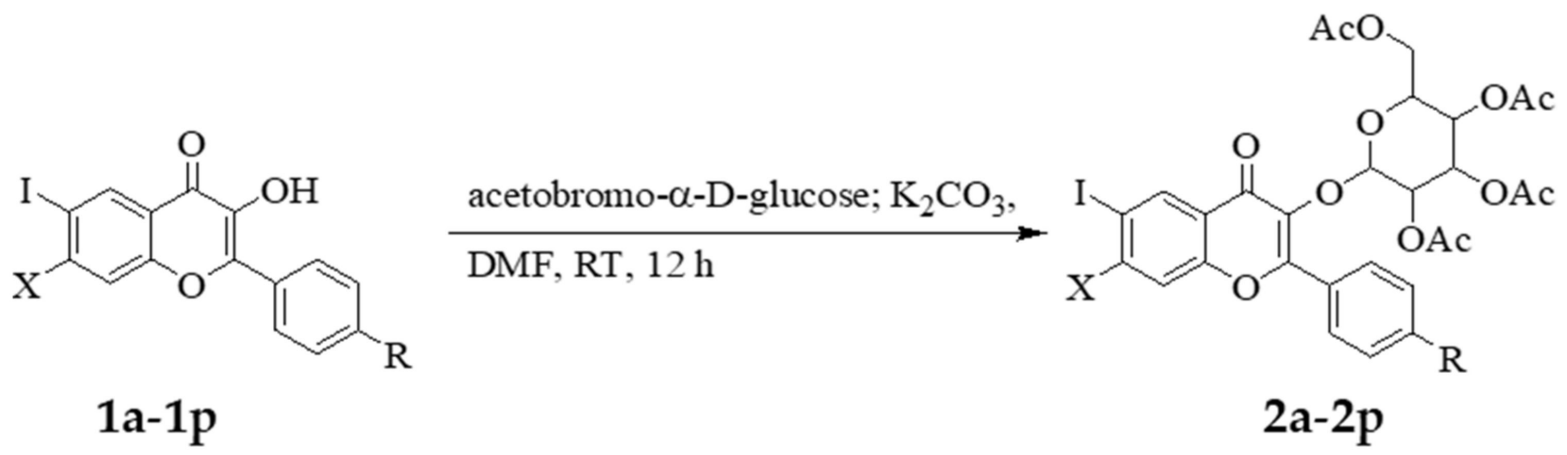
Synthesis of the 7-substituted 6-iodo-3-*O*-flavonol glycosides **2a**–**2p**.

**Figure 1:**
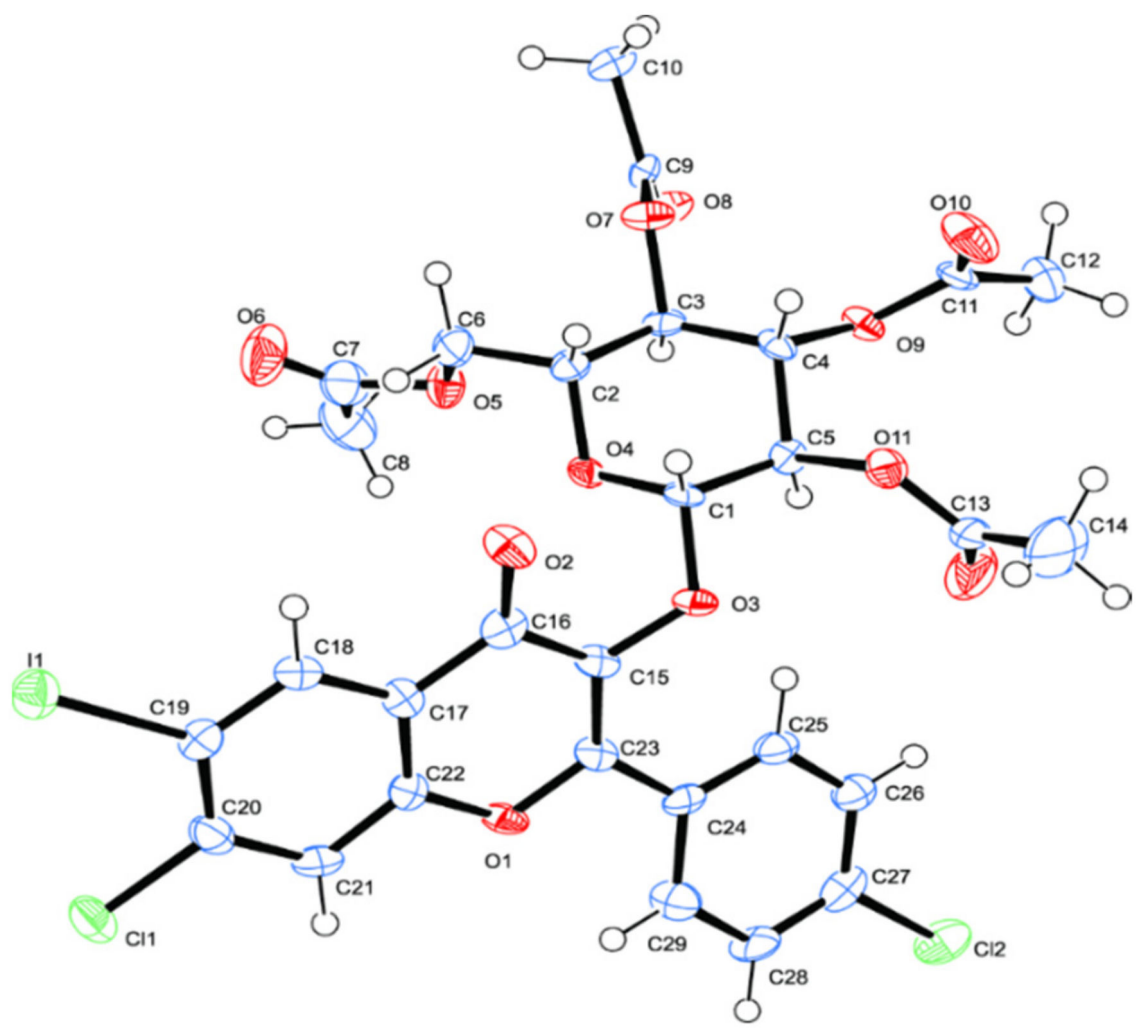
Oak Ridge Thermal Ellipsoid Plot (ORTEP) diagram (50% probability level) of **2g**.

The route used for the synthesis of the title compounds is outlined in Scheme 1 and it involved subjecting the known flavonols **1a**–**1p** [22] to glycosylation with acetobromo-α-d-glucose. The acylated 3-*O*-glycosides **2a**–**2p** (see Table 1 for the substitution pattern of these compounds) were purified by column chromatography on silica gel and then characterized using a combination of nuclear magnetic resonance (NMR), infrared (IR,) and mass spectrometric techniques. Their ^1^H-NMR spectra revealed the presence of signals in the aromatic region and additional peaks in the aliphatic region δ 3.60–5.70 ppm with two intense signals around δ 1.99 ppm and δ 2.02 ppm corresponding to the non-equivalent acetyl groups of the incorporated acylated sugar moiety. Moreover, their ^13^C-NMR spectra also revealed two characteristic signals around δ 173 ppm and δ 170 ppm corresponding to the 4-carbonyl and acetyl groups, respectively. Their IR spectra, on the other hand, lacked the absorption band for the 3-hydroxyl group and revealed the presence of additional carbonyl band around ν_max_ 1720 cm^−1^ for the acyl groups. The molecular masses of these compounds were confirmed by electron spray ionization mass spectrometry and these complementary spectroscopic techniques corroborated the assigned structures. Replacement of the 3-hydroxyl group with the acetylated 3-*O*-glycoside function on the flavone framework is, in fact, evident in the single crystal X-ray diffraction structure of compound **2g** shown in Figure 1 [23].

Compounds **2a**–**2p** were in turn, evaluated for potential biological properties as inhibitors of cholinesterase (AChE and/or BChE) and β-secretase (BACE-1) activities.

### 2.2. Inhibitory Activity of the 3-O-flavonol Glycosides Against AChE, BChE, and β-Secretase

Despite an increase in the use of drugs for receptors to modulate signals from outside the cell, 47% of all current drugs have been found to inhibit enzyme targets [24]. As a result, enzymes remain the primary targets for drug design because altering the activity of an enzyme has immediate and defined effects. Consequently, the 7-substituted 6-iodo-3-*O*-flavonol glycosides **2a**–**2p** were evaluated for inhibitory effect against acetylcholinesterase (AChE) and butyrylcholinesterase (BChE) activity using the known non-competitive reversible AChE inhibitor, donepezil, as a reference standard. Compounds **2a**–**2p** have been grouped into four series, namely **2a**–**2d** (X = F), **2e**–**2h** (X = Cl), **2i**–**2l** (X = Br) and **2m**–**2p** (X = OCH_3_) with the 2-aryl substituent representing C_6_H_5_–, 4-FC_6_H_4_–_,_ 4-ClC_6_H_4_–, and 4-MeOC_6_H_4_–, respectively (Table 2). The structure activity relationship (SAR) of these compounds has been rationalized with respect to the nature of substituent on the 2-phenyl substituent and the 7-position of the chromenone (flavone) moiety. The effect of *O*-glycoside moiety, on the other hand, has been rationalized by comparison with the previously published data of the corresponding flavonol precursors **1a**–**1p** [22]. A combination of fluorine atom at position-7 of the flavone skeleton with *O*-glycoside moiety in compounds **2a**–**2d** resulted in reduced or lack of inhibitory effect against both AChE and BChE activities. Significantly reduced inhibitory effect was observed for **2b** (IC_50_ = 28.2 ± 0.02 µM) and **2c** (IC_50_ = 24.74 µM) against AChE activity. The corresponding flavonol precursors **1b** (IC_50_ = 3.23 µM) and **1c** (IC_50_ = 3.35 µM), on the other hand, have previously been found to exhibit increased inhibitory effect against this enzyme, more so than donepezil (IC_50_ = 4.77 µM) at the same concentration [22]. Reduced inhibitory effect against AChE activity has also been observed for the 7-chloro substituted derivatives **2e**–**2h** though these compounds were moderately inhibiting against BChE activity when compared to the activity of donepezil (IC_50_ = 4.82 µM). A combination of 7-chloro and 2-(4-chlorophenyl)- group in the flavonol-3-*O*-glycoside **2g**, for example, resulted in significant inhibitory effect against BChE activity with IC_50_ value of 7.15 µM compared to the activity of donepezil. The activity of compound **2g** against the two enzymes was found to be comparable to the activity of the parent flavonol **1g**, which was previously found to exhibit reduced inhibitory effect against AChE (IC_50_ = 59.10 µM) and significant activity against BChE (IC_50_ = 7.69 µM) [22]. The presence of 3-*O*-glycoside moiety in **2h**, on the other hand, resulted in significantly reduced inhibitory effect against BChE activity (IC_50_ = 10.10 µM) when compared to the activity of the corresponding substrate **1h** (IC_50_ = 5.72 µM) [22]. The presence of bromine atom at position-7 resulted in moderate to increased inhibitory effect against AChE activity for compounds **2i**–**2l**. Within this series of compounds, the trend in activity increases with increasing size of the substituent at the *para* position of the 2-phenyl ring, H < F < Cl < OCH_3_, and is in the order 70.56 (**2i**) < 31.20 (**2j**) < 5.18 (**2k**) < 5.05 µM (**2l**). Hitherto, the size of the 2-aryl substituent showed an opposite effect on the anti-acetylcholinesterase activity of the corresponding substrates **1i**–**1l** [22]. A combination of 7-bromo and either 2-(4-chlorophenyl)- group in **2k** or a 2-(4-methoxyphenyl)- group in **2l** resulted in significant inhibitory effect against AChE activity than any of the compounds tested in this investigation. Compound **2k** seems to be selective towards AChE since it displayed reduced anti-BChE activity. Compound **2l**, on the other hand, appears to exhibit dual activity against both enzymes, though more so for AChE (IC_50_ = 5.05 µM) than BChE (IC_50_ = 7.24 µM) when compared to donepezil with IC_50_ values of 4.82 and 6.01 µM, respectively. The corresponding 3-flavonol precursors **1k** and **1l** were previously found to exhibit reduced inhibitory effect against the AChE activity (IC_50_ values of 29.4 and 28.99 µM, respectively), but increased inhibitory effect against BChE activity (5.25 and 4.88 µM, respectively) [22]. Within the series **2i**–**2l**, only compounds **2i** and **2l** exhibited significant inhibitory effect against BChE activity. The presence of a halogen atom at the *para* position of the 2-aryl group of flavones **2j** and **2k**, on the other hand, resulted in significantly reduced activity against this enzyme. For the series **2m**–**2p** substituted with a strongly pi-electron donating and lipophilic methoxy group at the 7-position, significant inhibitory effect against AChE activity was observed for derivatives **2n** and **2p** substituted with a 2-(4-fluorophenyl)- and 2-(4-methoxyphenyl)- group with IC_50_ values of 6.33 and 9.19 µM, respectively. The 2-(4-methoxyphenyl)–substituted derivative **2p** seems to exhibit dual activity against both enzymes, more so against BChE (IC_50_ = 5.21 µM) than AChE (IC_50_ = 9.19 µM). Hitherto, the corresponding substrate **1p** was found to exhibit significant inhibitory effect against AChE (IC_50_ = 7.19 µM) and BChE (IC_50_ = 3.29 µM) activities [22]. The modest selectivity values for compounds **2l** and **2p** (Table 2) may make these compounds potential dual inhibitors against AChE and BChE activities. Dual inhibition of AChE and BChE has become an efficient strategy to alleviate Alzheimer’s disease symptoms with minimal or no side effects [25].

High levels of AChE or BChE have been found to have a role in amyloid beta(Aβ)-peptide aggregation during the early stages of senile plaque formation as well as in other pathological characteristics of AD [26,27]. β-Site amyloid precursor protein (APP) cleaving enzyme 1 (BACE-1) plays a significant role in the cleavage of amyloid precursor protein, which leads to the production of α,β-peptide [28]. Overproduction of Aβ by β-secretase results in toxic fibrils causing neurodegeneration characteristic of the Alzheimer’s disease [29]. β-Secretase is considered the initial and rate-limiting step in Aβ production, which makes it another target for the treatment and prevention of AD. Certain flavonols and flavones have been reported to act as β-secretase inhibitors and to suppress β-secretase expression [13]. With the aim to discover compounds that could target different pathological features of the Alzheimer’s disease, we decided to evaluate compounds **2l** and **2p** with potential dual inhibitory effect against AChE and BChE activities for inhibitory effect against β-secretase activity. Quercetin has been employed as a reference standard for this assay because it has previously showed significant inhibitory effect against β-secretase and reduced the levels of Aβ in neurons [30]. Compounds **2l** and **2p** were found to be moderately inhibiting against β-secretase activity when compared to quercetin (IC_50_ = 13.7 µM) with IC_50_ values of 18.3 µM and 25.2 µM, respectively (Table 3). The inhibitory effect of these compounds against β-secretase activity are slightly higher than those previously observed for the corresponding flavonol precursors **1l** (IC_50_ = 15.74 µM) and **1p** (IC_50_ = 22.44 µM). Compounds **1l** and **1p** were previously found to be less inhibiting against this enzyme when compared to quercetin [22].

Since some of the glycosides retain the potent and selective cholinesterase and β-secretase inhibitory activity of the parent 3-flavonols, we decided to elucidate their plausible mechanisms of inhibition. Based on the in vitro results of ChE and β-secretase inhibition, the kinetic study was carried on compounds **2l** and **2p** as described below.

### 2.3. Kinetic Studies Against Cholinesterases and β-Secretase

Compounds **2l** and **2p** were subjected to kinetic studies at increasing concentrations (0.1, 0.5, 2.5 and 5.0 µM) of the substrates, acetylthiocholine iodide (ATChI) and butyrylthiocholine iodide (BTChI), respectively. The Lineweaver–Burk plots were drawn to determine the type of inhibition these compounds display against AChE and BChE (refer to Figure S2. In the Supplementary Material for the corresponding graphs). For compound **2l** there is a decrease in the Vmax as the inhibitor concentration is increased (0.054 to 0.018) while the Km remains relatively unchanged (0.16 ± 0.02 µM). This would indicate that **2l** inhibits the enzyme in a non-competitive manner binding to the enzyme and still allowing the substrate to bind. Analysis of the Lineweaver–Burk plot for **2p** also indicates non-competitive inhibition as there is a decrease in the Vmax of AChE (0.033 to 0.015 for 0 to 5 µM of **2p**, respectively) while Km remains relatively unaffected 0.13 ± 0.03 µM with increasing inhibitor concentrations (0 to 5 µM **2p**). Both compounds seem to display non-competitive inhibition towards BChE. The Vmax values for **2l** decrease from 0.064 to 0.017 while Km remains unchanged (0.19 ± 0.03 µM) with increasing inhibitor concentrations from 0 to 5µM. Similarly for **2p** the Vmax values decrease from 0.057 to 0.016 while Km remains unchanged (0.22 ± 0.01 µM) with increasing inhibitor concentrations from 0 to 5µM. Dixon plots for **2l** and **2p** against both AChE and BChE were used to determine the Ki or inhibition constant values for the two compounds (see Figure S2 in the Supplementary Material). The inhibition constants (Ki) against AChE were determined to be 1.38 ± 0.62 and 2.36 ± 0.32 for compounds **2l** and **2p**, respectively. Ki values for compounds **2l** and **2p** against BChE were calculated to be 1.51 ± 0.04 and 1.62 ± 0.03, respectively. Kinetic studies were also conducted to determine the mode of inhibition exhibited by compounds **2l** and **2p** against β-secretase (refer to Figure S3 in the Supplementary Material). Based on Lineweaver–Burk plots and resulting Km and Vmax values compound **2l** displays non-competitive inhibition with unchanged Km values (0.20 ± 0.1) in the absence and presence of increasing inhibitor concentration while the Vmax values decrease from 0.04 to 0.02. Similarly for **2p**, the Km values remain relatively unchanged (3.6 ± 0.2 µM) while Vmax decreases from 0.09 in the absence of inhibitor to 0.02 in the presence of 5 µM inhibitor. Therefore, **2p** displays non-competitive inhibition of β-secretase. Ki values obtained from Dixon plots for the inhibition of β-secretase by **2l** and **2p** were calculated to be 2.42 ± 0.05 and 2.94 ± 0.02, respectively.

### 2.4. Molecular Docking Studies into AChE, BChE and β-Secretase Active Sites

To rationalize the SAR and establish plausible protein–ligand interactions at molecular level, we docked the most active compounds into the active pockets of AChE (compounds **2k**, **2l**, **2n**, and 2p), BChE (compounds **2e**, **2g**, **2l** and **2p**) and β-secretase (compounds **2l** and **2p**).

#### 2.4.1. Molecular Docking Studies of Compounds **2k**, **2l**, **2n**, and **2p** into AChE Active Sites

**Figure 2:**
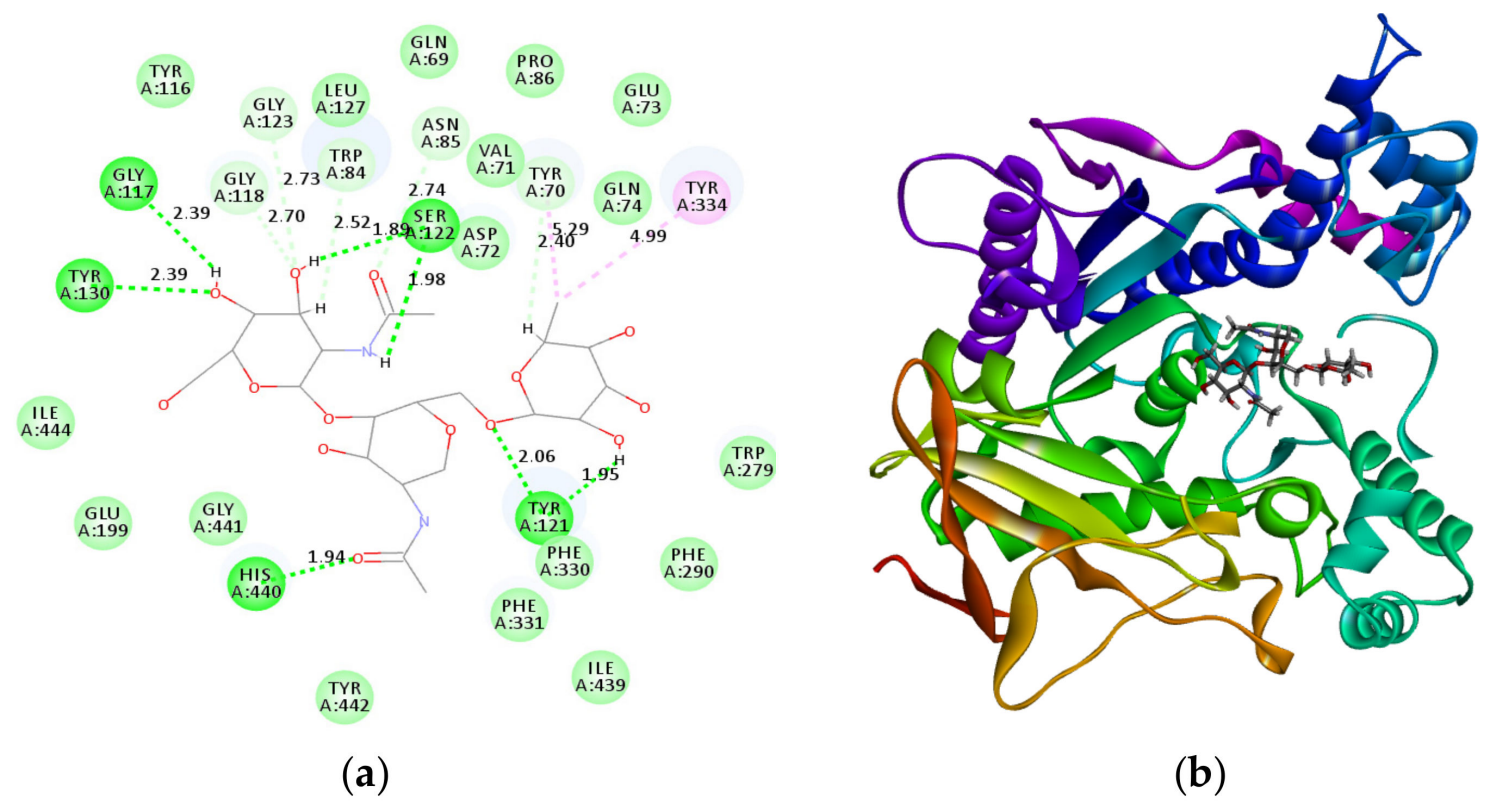
Docking pose of donepezil (**a** & **b**) with AChE (Protein Data Bank (PDB): 1GQR). Interactions are color coded: bright green represents conventional hydrogen bonds; light green: van der Waals interactions; very light green: carbon hydrogen bonds; dark pink: pi–pi interactions; and light pink: pi–alkyl and alkyl interactions.

Compounds **2k**, **2l**, **2n**, and **2p** were docked into the active pockets AChE against donepezil (Figure 2a,b), which is known to interact with both the catalytic active site (CAS) and the peripheral anionic site (PAS) tryptophans via ring-stacking interactions [31].

**Figure 3:**
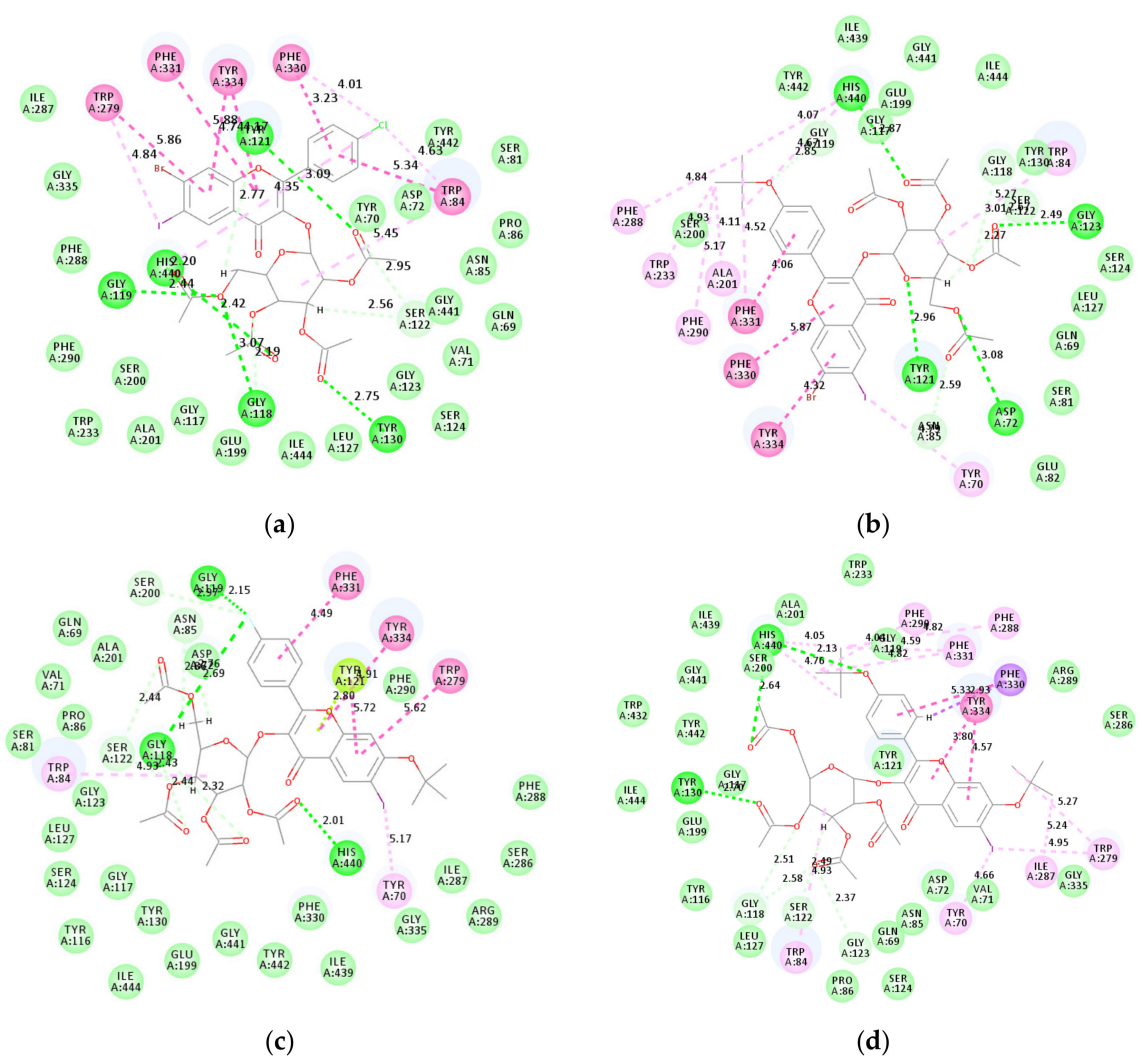
Docking poses of **2k** (**a**), **2l** (**b**), **2n** (**c**), and **2p** (**d**) into AChE. Bright green represents conventional hydrogen bonds; light green: van der Waals interactions; very light green: carbon hydrogen bonds; dark pink: pi–pi interactions; light pink: pi–alkyl interactions; purple: pi-sigma interactions; and green yellow: pi–lone-pair interactions.

Figure 3 shows the docking poses of compounds **2k**, **2l**, **2n**, and **2p** into AChE active sites. The flavone framework of compound **2k** (Figure 3a) is involved in pi–pi stacking and pi–pi T-shaped interactions with the protein residues Trp84, Trp279, Tyr334, Phe330, and Phe331. Similar interactions are predicted between the flavone moiety of **2l** (Figure 3b) and the protein residues Tyr334, Phe330, and Phe331. Chlorine atom of **2k** is involved in alkyl and pi–alkyl interactions with the protein residues Phe330, Trp84, and His440, whereas its iodine atoms exhibits similar interactions with Trp279. Molecular docking predicts several hydrogen bond interactions between the glycoside moiety of **2k** and the protein residues Tyr121, Tyr130, Gly118 and Gly119 as well as histidine-440 (His440, Hb distance = 2.19 Å) of the catalytic triad. Compound **2l**, on the other hand, is involved in hydrogen bond interactions with Asp72, Tyr121 and Gly123 as well as His440 (Hb distance = 2.20 Å). Additional alkyl and pi–alkyl interactions are predicted between the methoxy group of **2l** and the protein residues Trp233, Ala201, Phe290 and His440. Both molecules seem to penetrate the aromatic cleft of the AChE through chromone scaffold and their entrance into the aromatic cleft is supported by pi–pi interaction with phenylalanine (Phe330) in the acyl binding pocket. The observed slight increase in inhibitory effect of **2l** against AChE activity is presumably due to the increased lipophilicity of the methoxy group, which seems to interact with more protein residues than chlorine atom of **2k**. The flavone framework of **2n** (Figure 3c), on the other hand, is involved in pi–pi stacking and pi–pi T-shaped interactions with Tyr334, Trp279, and Phe331. Pi–lone-pair interaction is predicted between ring-C and the protein residue, Tyr121. Hydrogen bond interactions are predicted between the glycoside arm and the protein residues Gly118 and His440. Gly118 and Gly119 are also involved in hydrogen bonding with the methoxy group of the 2-aryl substituent. These increased interactions probably account for the significant inhibitory effect of this compound against AChE activity (IC_50_ = 6.33 µM). The flavone framework, iodine atom and the methoxy group of compound **2p** (Figure 3d), are involved in hydrophobic interactions with several protein residues. Hydrogen bond interactions are predicted between the glycoside moiety and the protein residues Tyr130 and His440.

Compounds **2k**, **2l**, **2n**, and 2p are predicted to have hydrophobic interactions with protein residues Trp279 and Trp334 in the PAS of AChE, and noncovalent interactions (hydrogen bonding, van der Waals interactions and pi stacking, etc.) with His440 of the catalytic triad. These interactions which involve both the flavone scaffold and the glycoside wing probably account for the observed increased inhibitory effect of the compounds against the AChE activity. The predicted interactions of these compounds with protein residues in the CAS and PAS indicate that binding can take place at the active site of these enzymes. The active site cleft is large, and the non-competitive mode of inhibition displayed by compounds **2l** and **2p** would indicate that their binding does no exclude substrate binding at the active sites. The binding presumably affects the enzyme’s activity by altering its conformation and affecting its catalytic activity.

#### 2.4.2. Molecular Docking Studies of **2e**, **2g**, **2l**, and **2p** into BChE Active Sites

**Figure 4:**
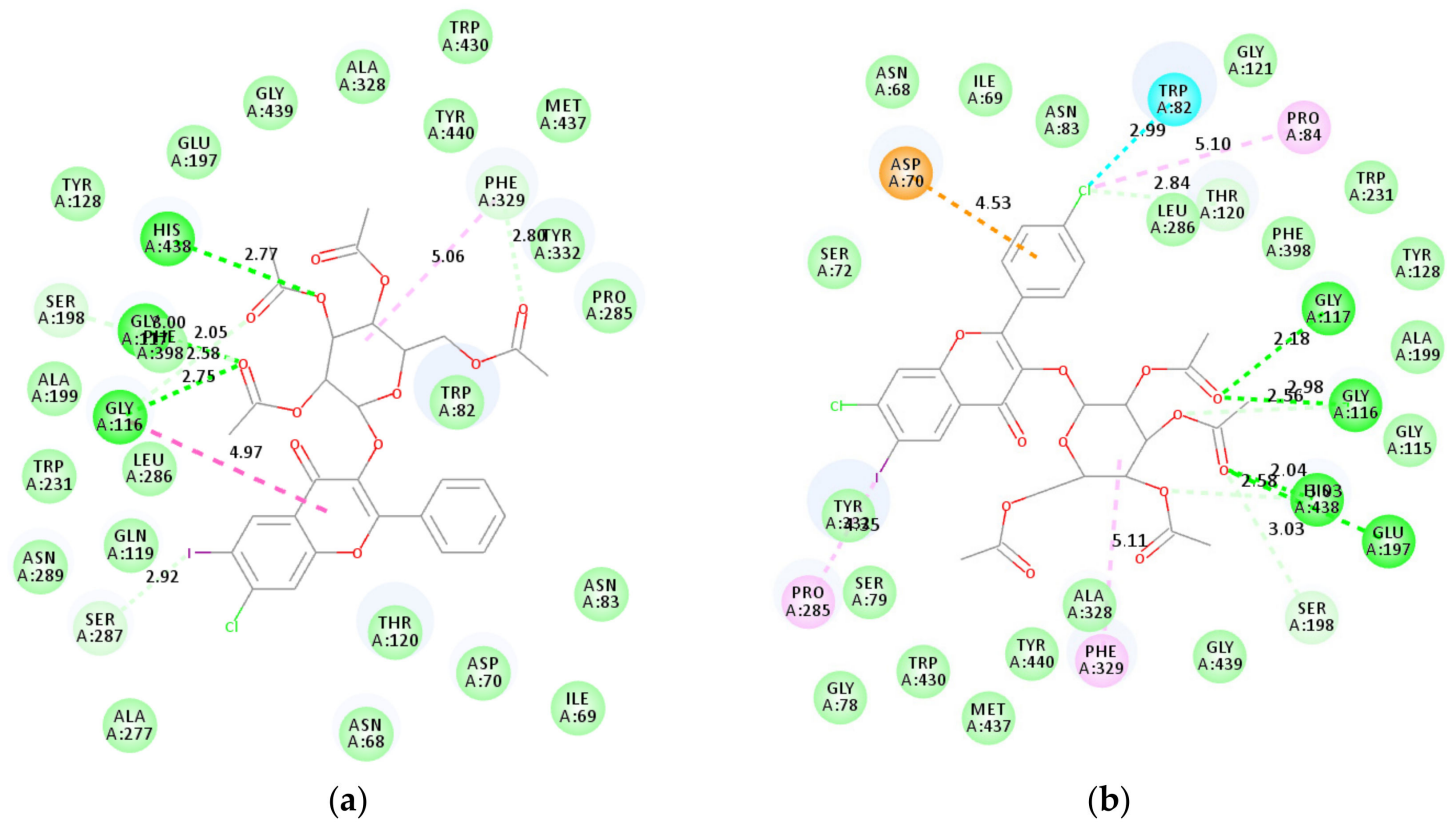

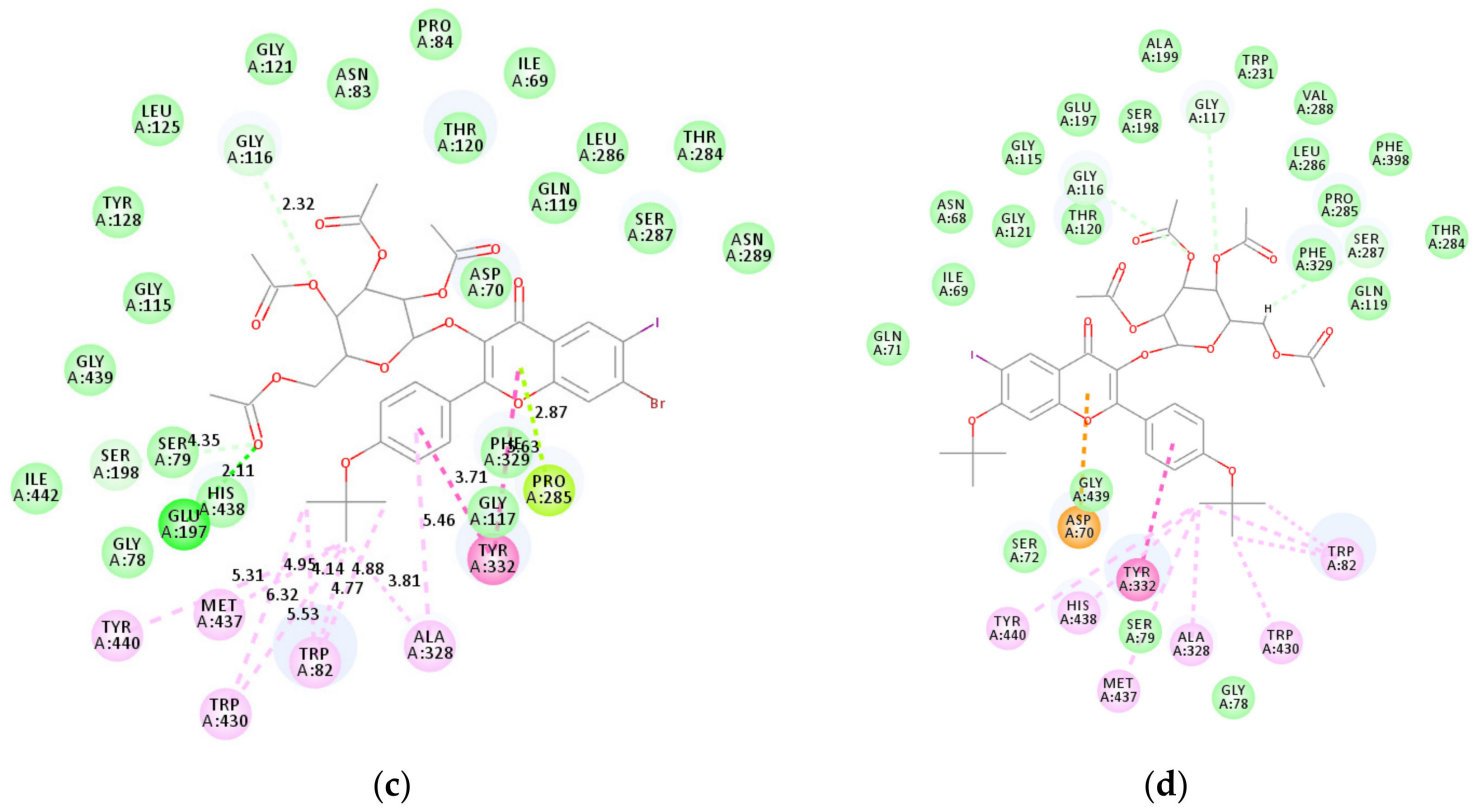
Docking poses of compounds **2e** (**a**), **2g** (**b**), **2l** (**c**), and **2p** (**d**) into BChE. Bright green represents conventional hydrogen bonds; light green: van der Waals interactions; very light green: carbon hydrogen bonds; orange: pi–anion interaction; light blue: halogen bonds; dark pink: pi–pi interactions; and light pink: pi–alkyl and alkyl interactions.

The docking poses of compounds **2e**, **2g**, **2l, and 2p** into BChE active sites (Protein Data Bank (PDB): 1P0I) are represented in Figure 4. Weak van der Waals and carbon hydrogen bond interactions exist between the glycoside ring of compound **2e** (Figure 4a) with Gly116 and Thr120. Ring-A and ring-B of this compound are involved in pi–pi and amide-pi interaction with Ile69 and Tyr332, respectively. Its C-ring, on the other hand, is involved in pi-anion interaction with Asp70, while pi–alkyl interaction is predicted between ring-B and Ala328. Hydrogen bond interactions are predicted to exist between the carbonyl oxygen atoms of the acetoxy groups of the glycoside fragment with Gly117, Glu197, Tyr128, and His438. These increased interactions presumably account for the observed moderate inhibitory effect of **2e** against the BChE activity. Chlorine atom on ring-B of **2g** (Figure 4b) is involved in halogen bonding with Trp82 at the choline-binding site and there is also pi-anion interaction between this ring and Asp70. Alkyl and pi–alkyl interactions exist between chlorine and Pro84, and between iodine and Pro285 as well as between the glycoside ring and Phe329. There are several hydrogen bond interactions involving the glycoside moiety and the protein residues Gly116, Gly117, Glu197, and His438. Additional interactions of the 4-chlorophenyl group with Trp82 and Asp70 via halogen bonding and pi-anion interaction presumably account for the observed increased inhibitory effect of **2g** (IC_50_ = 7.15 µM) compared to **2e** (IC_50_ = 9.03 µM). The lipophilic 2-(4-methoxyphenyl) group of the most active compound **2l** (Figure 4c) against AChE activity is involved in alkyl and pi–alkyl interactions with several protein residues. Tyr332 is involved in pi–pi stacking interaction with the B- and C-rings and there is also a pi–lone-pair interaction between protein residue Pro285 and the C-ring. Hydrogen bond interaction is predicted between the carbonyl oxygen of the methyl acetate group with Glu197 and this oxygen atom is also involved in weak van der Waals interaction with Ser79. The only other weak interaction involving carbon hydrogen bond interaction exists between Gly116 and one of the acetoxy groups. Although the inhibitory effects of **2l** (IC_50_ = 7.24 µM) and **2g** (IC_50_ = 7.15 µM) are comparable, the latter is slightly more active presumably because of halogen bond interaction between Cl and Trp82. Compound **2p** (Figure 4d), on the other hand, has been found to be the most inhibiting against BChE activity among the title compounds. Its C-ring is involved in pi-anion interaction with Asp70 while the glycoside moiety is involved in weak carbon hydrogen bond and van der Waals interactions with several protein residues. Several hydrophobic (alkyl, pi–alkyl and pi–pi stacking) interactions are predicted between the lipophilic 2-(4-methoxyphenyl) group of compound **2p** with the protein residues Trp82, Trp430, Ala328, Met437, and Tyr440 including His438. No hydrogen bond interactions have been predicted between this compound and any of the protein residues. The predicted interaction of these compounds with His438 in the catalytic active site probably accounts for their significant inhibitory effect against BChE activity. These compounds might also bind additional alternative sites on the enzyme consistent with their non-competitive inhibition mode. Binding at alternative sites would alter the enzyme’s conformation and hence its catalytic activity.

Substrates **1l** and **1p** were previously predicted to form hydrogen bonds with His438 and Ser198 of the catalytic triad of BChE [22] consisting of serine (Ser198), histidine (His438) and glutamate (Glu325) interconnected by hydrogen bonds [32]. The predicted interaction of the glycoside derivatives **2l** and **2p** only with His438 would probably indicate that these compounds bind BChE, but do not compete with the substrate. This would allow the substrate to bind to the active site and probably account for the non-competitive mode of action confirmed by the kinetic studies above.

#### 2.4.3. Molecular Docking Studies of Compounds **2l** and **2p** into β-Secretase Active Sites

**Figure 5:**
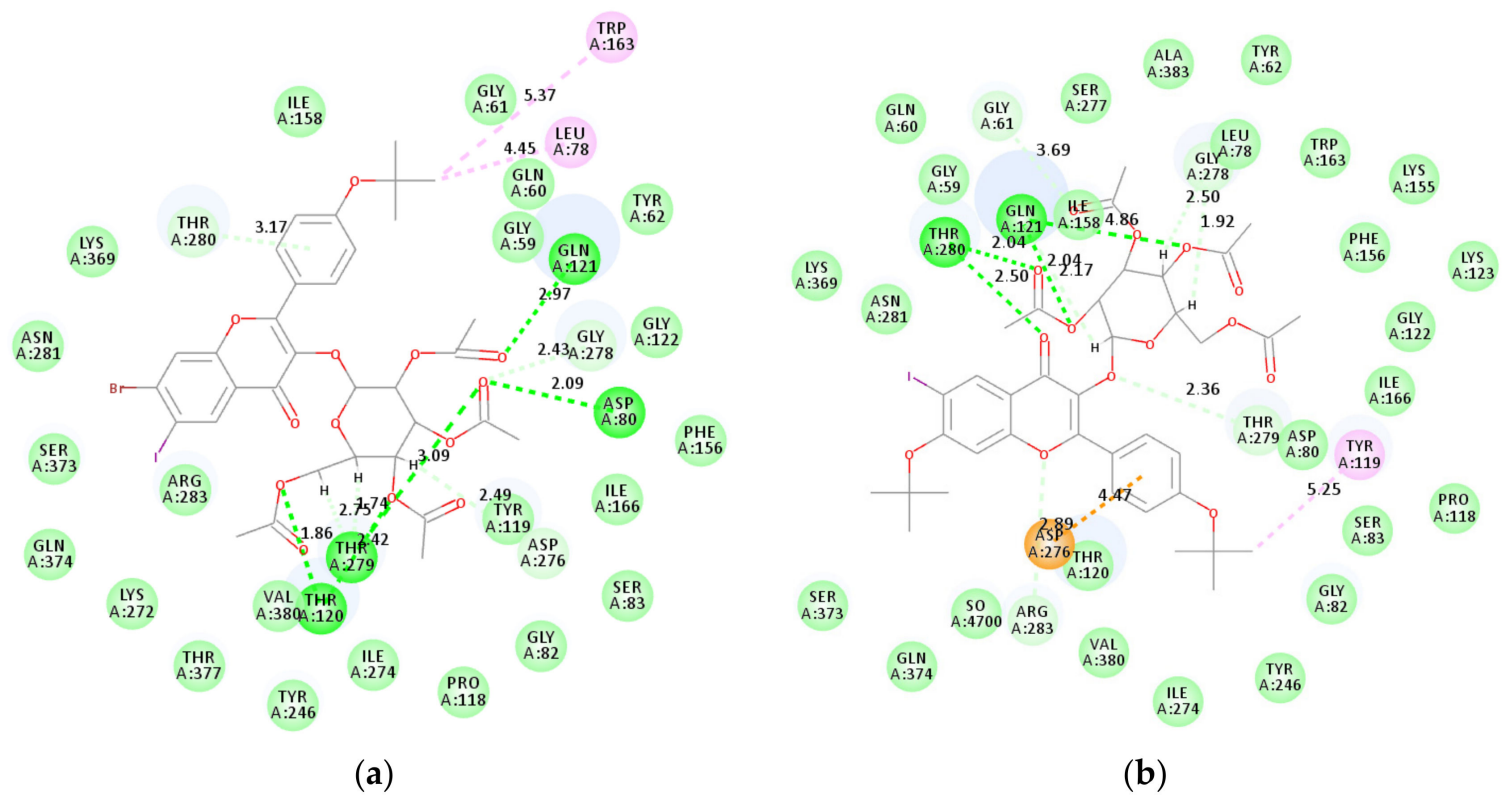
Docking poses of **2l** (**a**) and **2p** (**b**) into β-secretase. Bright green represents conventional hydrogen bonds; light green: van der Waals interactions; very light green: carbon hydrogen bonds; orange: pi–anion interaction; dark pink: pi–pi interactions; and light pink: pi–alkyl and alkyl interactions.

β-Secretase has proven to be a difficult target compared to cholinesterase due to its extended active site and inherent flexibility. The catalytic domain of β-secretase, for example, contains eight pockets consisting of different amino acid residues [33] and different inhibitors can bind to different sites and a few sites simultaneously [34]. Both compounds **2l** (Figure 5a) and **2p** (Figure 5b) were docked into β-secretase (PDB 3IXJ) and they are predicted to bind better to the S1′ pocket (Figure 5), which accommodates large groups. This pocket is close to the active site comprising of the catalytic aspartate dyad, Asp32 and Asp228. The two compounds exhibit several key protein–ligand interactions including hydrogen bond between ester functions of the glucoside moiety with the protein residues Asp80, Gln121, Thr120, Thr279, and Thr280. Molecular docking predicts several hydrogen bond interactions for compound **2l** than **2p**, and this correlates with the observed IC_50_ values. However, the two compounds show no interaction with the catalytic aspartic acids, Asp32 and Asp228. This is consistent with the observed non-competitive mode of inhibition, which indicates that the binding of these compounds affects the proteins structure and the enzyme’s ability to catalyze the reaction. The compounds seem not to bind to the active site, instead, they probably alter the conformational dynamics of the protein affecting catalytic activity. Molecular docking helped to explain the observed inhibitory activity, selectivity, and mechanism of cholinesterase inhibition by these compounds. The docking studies of the title compounds into the ChE and β-secretase active sites revealed increased interaction of the glycoside moiety with several protein residues.

Chromones and their flavonoid derivatives are known to play important roles as antioxidants and radical scavengers [14,35]. The design of anticholinesterase inhibitors with antioxidant properties, on the other hand, continues to attract considerable attention towards efficient therapy of AD. This MTDL design strategy has been found to be more effective in the treatment of Alzheimer’s disease than the use of single-targeted drugs [9,10,11]. Consequently, in the last part of this investigation, we evaluated the most active compounds against AChE and/or BChE for antioxidant properties.

### 2.5. Antioxidant Activity of Compounds ***2g***, ***2k***, ***2l***, ***2n***, and ***2p***

The free-radical scavenging activities of compounds **2g**, **2k**, **2l**, **2n, and 2p** were evaluated against the natural antioxidants ascorbic acid and quercetin [36] as reference standards. The results are expressed as IC_50_ values, i.e., the concentration of each sample required or able to scavenge 50% of the DPPH (Table 4). Preliminary 2,2-diphenyl-1-picrylhydrazyl (DPPH) radical scavenging results revealed that the most active glycosides against AChE and/or BChE activity exhibit moderate antioxidant activity (IC_50_ 11.4–21.6 µM) when compared to the reference standards, ascorbic acid (IC_50_ = 5.5 µM) and quercetin (IC_50_ = 3.1 µM). A combination of the electron-withdrawing 2-(4-chlorophenyl) group and a chlorine or bromine atom at the 7-position of the chromone framework of compounds **2g** and **2k** resulted in significant radical scavenging abilities with IC_50_ values 13.1 µM and 11.4 µM, respectively. Compounds **2g** and **2k** exhibit increased inhibitory effect against AChE activity and free-radical scavenging ability. A combination of a strongly electron donating 2-(4-methoxyphenyl) group and an electron-withdrawing 7-bromo atom on the framework of **2l**, on the other hand, resulted in relatively reduced radical scavenging ability (IC_50_ = 21.6 µM). On the other hand, a combination of moderately 2-(4-fluorophenyl) and strongly electron delocalizing 7-methoxy groups resulted in significant free-radical scavenging ability for compound **2n**. Compounds **2l** and **2p** with potential dual inhibitory activity against AChE and BChE exhibited moderate free-radical scavenging ability than the other derivatives. However, the presence of strongly electron donating 7-methoxy group in compound **2p** resulted in higher free-radical scavenging ability than **2l** substituted with a bromine atom at this position. Although compound **2p** exhibited relatively reduced activity against BACE-1, it has potential to serve as a dual AChE and BChE inhibitor with antioxidant potential.

## 3. Materials and Methods

### 3.1. General

Merck kieselgel 60 (0.063–0.200 mm) (Merck KGaA, Frankfurt, Germany) was used as stationary phase for column chromatography. The melting point (m.p.) values of the prepared compounds were recorded on a Thermocouple digital melting point apparatus and are uncorrected. NMR spectra were recorded using Varian Mercury 300 MHz NMR spectrometer (Varian Inc., Palo Alto, CA, USA) and Agilent 500 MHz NMR spectrometers (Agilent Technologies, Oxford, UK) and the chemical shifts are quoted relative to tetramethylsilane (TMS) peak. IR spectra were recorded by thin-film method using a Bruker VERTEX 70 FT-IR Spectrometer (Bruker Optics, Billerica, MA, USA) fitted with a diamond attenuated total reflectance (ATR) accessory. High-resolution mass spectra were recorded using Waters Synapt G2 Quadrupole Time-of-flight mass spectrometer (Waters Corp., Milford, MA, USA). The synthesis and analytical data of flavonols **1a**–**1p** have been described before [22].

### 3.2. Typical Procedure for the Synthesis of the 7-substituted-6-iodo-2-phenyl-chromen-4-one-3-O-2,3,4,6-O-tetraacetyl-β-d-glucopyranoside Derivatives (***2a**–**2p***)

A mixture of **1a** (0.20 g, 0.50 mmol), acetobromo-α-d-glucose (0.25 g, 0.60 mmol) and K_2_CO_3_ (0.08 g, 0.60 mmol) in DMF (20 mL) was stirred at room temperature for 12 h. The reaction was poured onto crush ice. The organic phase was extracted with chloroform and the combined organic layers were dried over anhydrous MgSO_4_. The solvent was evaporated on a rotary evaporator under reduced pressure. The residue was purified on silica gel column chromatography to afford **2a**. Compounds **2a**–**2p** were prepared in this fashion

*7-Fluoro-6-iodo-2-phenyl-4H-chromen-4-one-3-O-2,3,4,6-O-tetraacetyl-β-d-glucopyranoside* (**2a**): White solid (0.25 g, 71%), R*_f_* (50% EtOAc‒hexane) 0.67, m.p. 219–220 °C; ν_max_ (ATR) 433, 578, 761, 1032, 1186, 1209, 1421, 1640, 1758, 2614, 3062, 3077 cm^−1^; ^1^H-NMR (300 MHz, DMSO-*d*_6_) 1.85 (3H, s, -C(O)CH_3_), 1.94 (3H, s -C(O)CH_3_), 1.96 (3H, s, -C(O)CH_3_), 2.03 (3H, s, -C(O)CH_3_), 3.87–3.94 (3H, m, aliphatic), 4.89–5.00 (2H, m, aliphatic), 5.39 (1H, t, *J* = 10.0 Hz, aliphatic), 5.75 (1H, d, *J* = 7.5 Hz, aliphatic), 7.54–7.57 (3H, m, H-4′ and H-3′,5′), 8.04 (2H, d, *J* = 4.8 Hz, H-2′ and 6′), 8.14 (1H, s H-8), 8.47 (1H, s, H-5); ^13^C-NMR (75 MHz, DMSO-*d*_6_) 20.7, 20.8, 20.9, 61.6, 68.3, 71.1, 71.2, 72.2, 79.6 (d, ^2^*J*_CF_ = 27.4 Hz), 105.4 (d, ^2^*J*_CF_ = 28.6 Hz), 120.7, 128.0 (2×C), 128.9 (2×C), 130.5 (2×C), 131.3, 135.7 (d, ^3^*J*_CF_ = 4.6 Hz), 140.0, 146.4, 155.7 (d, ^3^*J*_CF_ = 13.7 Hz), 163.6 (d, ^1^*J*_CF_ = 247.2 Hz), 169.7, 170.0, 170.1, 171.9; HRMS (ES^+^): *m*/*z* [M + H]^+^ calc for C_29_H_27_O_12_FI: 713.0531; found 713.0527.

*7-Fluoro-(4-fluorophenyl)-6-iodo-4H-chromen-4-one-3-O-2,3,4,6-O-tetraacetyl-β-d-glucopyranoside* (**2b**): White solid (0.25 g, 71%), R*_f_* (50% EtOAc‒hexane) 0.66, m.p. 254–255 °C; ν_max_ (ATR) 452, 542, 771, 1027, 1161, 1212, 1426, 1632, 1753, 2634, 3065, 3062 cm^−1^; ^1^H-NMR (300 MHz, DMSO-*d*_6_) 1.89 (3H, s, -C(O)CH_3_), 1.93 (3H, s, -C(O)CH_3_), 2.01 (3H, s, -C(O)CH_3_), 2.13 (3H, s, -C(O)CH_3_), 3.61 (1H, d, *J* = 10.5 Hz, aliphatic), 3.59‒3.98 (2H, m, aliphatic), 5.07 (1H, t, *J* = 9.6 Hz, aliphatic), 5.15–5.32 (2H, m, aliphatic), 5.65 (1H, d, *J* = 7.5 Hz, aliphatic), 7.01 (2H, d, *J* = 8.7 Hz, H-3′,5′), 7.75 (1H, d, *J* = 8.1 Hz, H-8), 8.13 (2H, d, *J* = 9.0 Hz, H-2′,6′), 8.42 (1H, d, *J* = 6.9 Hz, H-5); δ_C_ (75 MHz, CDCl_3_) 20.6, 20.7, 20.8, 21.0, 68.4, 71.1, 71.3, 73.3, 80.1 (d, ^2^*J*_CF_ = 26.3 Hz), 105.8 (d, ^2^*J*_CF_ = 26.3 Hz), 114.9 (d, ^2^*J*_CF_ = 24.0 Hz), 116.3 (d, ^2^*J*_CF_ = 21.75 Hz), 119.0 (d, ^4^*J*_CF_ = 22.8 Hz), 130.5 (d, ^4^*J*_CF_ = 3.4 Hz), 131.0 (d, ^3^*J*_CF_ = 8.025 Hz), 140.3 (d, ^4^*J*_CF_ = 4.5 Hz), 145.4, 164.5, 164.1, (d, ^1^*J*_CF_ = 251.9 Hz), 165.3 (d, ^1^*J*_CF_ = 249.4 Hz), 169.8, 170.0, 170.1, 171.3; HRMS (ES^+^): *m*/*z* [M + H]^+^ calc for C_29_H_26_O_12_F_2_I: 731.0437; found 731.0433.

*7-Fluoro-2-(4-Chlorophenyl)-6-iodo-4H-chromen-4-one-3-O-2,3,4,6-O-tetraacetyl-β-d-glucopyranoside* (**2c**): Brown solid (0.28 g, 77%), R*_f_* (50% EtOAc‒hexane) 0.72, m.p. 260–261 °C; ν_max_ (ATR) 465, 562, 768, 1029, 1163, 1214, 1430, 1635, 1754, 2640, 3065, 3071 cm^−1^; ^1^H-NMR (300 MHz, DMSO-*d*_6_) 1.80 (3H, s, -C(O)CH_3_), 1.95 (3H, s -C(O)CH_3_), 2.00 (3H, s, -C(O)CH_3_), 2.05 (3H, s, -C(O)CH_3_), 3.88‒3.97 (3H, m, aliphatic), 4.93–5.06 (2H, m, aliphatic), 5.40 (1H, t, *J* = 9.9 Hz, aliphatic), 5.75 (1H, d, *J* = 8.1 Hz, aliphatic), 7.07 (2H, d, *J* = 9.3 Hz, H-3′,5′), 7.77 (1H, d, *J* = 8.4 Hz, H-8), 8.07 (2H, d, *J* = 9.3 Hz, H-2′,6′), 8.40 (1H, d, *J* = 6.9 Hz, H-5); ^13^C-NMR (75 MHz, DMSO-*d*_6_) 20.6, 20.7, 20.8, 21.0. 61.6, 68.3, 71.0, 71.8, 72.2, 80.2 (d, ^2^*J*_CF_ = 28.6 Hz), 98.7, 106.2 (d, ^2^*J*_CF_ = 28.7 Hz),114.2, 122.3 (d, ^3^*J_CF_* = 12.6 Hz), 122.5, 131.3, 135.6, 136.1 (d, ^4^*J*_CF_ = 4.75 Hz), 155.9 (d, ^3^*J*_CF_ = 13.4 Hz), 157.4, 161.9, 163.9, (d, ^1^*J*_CF_ = 248.5 Hz), 169.7, 169.8, 170.0, 170.1, 171.4; HRMS (ES^+^): *m*/*z* [M + H]^+^ calc for C_29_H_26_O_12_F^35^ClI: 747.0142; found 747.0137.

*7-Fluoro-6-iodo-2-(4-methoxyphenyl)-4H-chromen-4-one-3-O-2,3,4,6-O-tetraacetyl-β-d-glucopyranoside* (**2d**): White solid (0.28 g, 80%), R*_f_* (50% EtOAc‒hexane) 0.63, m.p. 247–248 °C; ν_max_ (ATR) 472, 573, 760, 1024, 1161, 1204, 1423, 1638, 1748, 2656, 3079, 3077 cm^−1^; δ_H_ (500 MHz, DMSO-*d*_6_) 1.80 (3H, s, -C(O)CH_3_), 1.96 (3H, s -C(O)CH_3_), 2.00 (3H, s, -C(O)CH_3_), 2.05 (3H, s, -C(O)CH_3_), 3.85 (3H, s, -C(O)CH_3_), 3.86–3.96 (3H, m, aliphatic), 4.91–5.05 (2H, m, aliphatic), 5.41 (1H, t, *J* = 10.0 Hz, aliphatic), 5.76 (1H, d, *J* = 8.0 Hz, aliphatic), 7.09 (2H, d, *J* = 9.0 Hz, H-3′,5′), 7.80 (1H, d, *J* = 8.5 Hz, H-8), 8.08 (2H, d, *J* = 8.5 Hz, H-2′,6′), 8.39 (1H, d, *J* = 7.0 Hz, H-5); δ_C_^13^C-NMR (125 MHz, DMSO-*d*_6_) 20.6, 20.7, 20.8, 20.9, 55.9, 61.6, 68.4, 71.1, 71.8, 72.2, 80.2 (d, ^2^*J*_CF_ = 28.5 Hz), 98.7, 106.1 (d, ^2^*J_CF_* = 29.4 Hz), 114.1, 122.4 (d, ^3^*J*_CF_ = 16.1 Hz), 122.5, 131.2, 135.6, 136.0 (d, ^4^*J*_CF_ = 4.6 Hz), 156.0 (d, ^3^*J*_CF_ = 13.7 Hz), 157.5, 162.0, 163.8, (d, ^1^*J*_CF_ = 248.3 Hz), 169.7, 169.8, 169.9, 170.1, 171.3; HRMS (ES^+^): *m*/*z* [M + H]^+^ calc for C_30_H_29_O_13_FI: 743.0637; found 743.0637.

*7-Chloro-6-iodo-2-phenyl-4H-chromen-4-one-3-O-2,3,4,6-O-tetraacetyl-β-d-glucopyranoside* (**2e**): White solid (0.26 g, 73%), R*_f_* (50% EtOAc‒hexane) 0.67, m.p. 212–213 °C; ν_max_ (ATR) 438, 589, 772, 1036, 1194, 1214, 1425, 1645, 1750, 2611, 3064, 3078 cm^−1^; ^1^H-NMR (300 MHz, DMSO-*d*_6_) 1.89 (3H, s, -C(O)CH_3_), 2.02 (3H, s -C(O)CH_3_), 2.10 (3H, s, -C(O)CH_3_), 2.10 (3H, s, -C(O)CH_3_), 3.44–3.51 (2H, m, aliphatic), 3.63 (1H, d, *J* = 9.3 Hz, aliphatic), 5.07 (1H, t, *J* = 9.3 Hz, aliphatic), 5.15–5.31 (2H, m, aliphatic), 5.67 (1H, d, *J* = 7.8 Hz, aliphatic), 7.50 (3H, m, H-4′ and H-3′,5′), 7.69 (1H, s H-8), 8.00 (2H, d, *J* = 4.8 Hz, H-2′ and 6′), 8.68 (1H, s, H-5); ^13^C-NMR (75 MHz, DMSO-*d*_6_) 15.3, 20.5, 20.6, 20.8, 61.3, 65.9, 68.1, 71.5, 71.7, 72.6, 93.6, 98.8, 118.8, 123.8, 128.3, 129.1, 130.0, 131.2, 137.2, 143.7, 155.0, 157.7, 169.5, 169.8, 170.1, 170.3, 172.0; HRMS (ES^+^): *m*/*z* [M + H]^+^ calc for C_29_H_27_O_12_^35^ClI: 729.0236; found 729.0234.

*7-Chloro*-2-*(4-fluorophenyl)-6-iodo-4H-chromen-4-one-3-O-2,3,4,6-O-tetraacetyl-β-d-glucopyranoside* (**2f**): White solid (0.26 g, 66%), R*_f_* (50% EtOAc‒hexane) 0.68, m.p. 198–199 °C; ν_max_ (ATR) 469, 566, 777, 1039, 1154, 1213, 1430, 1643, 1754, 2642, 3071, 3076 cm^−1^; ^1^H-NMR (300 MHz, DMSO-*d*_6_) 1.90 (3H, s, -C(O)CH_3_), 2.00 (3H, s -C(O)CH_3_), 2.01 (3H, s, -C(O)CH_3_), 2.17 (3H, s, -C(O)CH_3_), 3.54–3.66 (1H, m, aliphatic), 3.83–4.12 (2H, m), 5.06 (1H, t, *J* = 9.5 Hz, aliphatic), 5.14–5.31 (2H, m, aliphatic), 5.65 (1H, d, *J* = 7.5 Hz, aliphatic), 7.84 (2H, dd, *J*_HH_ *=* 8.7 and *J*_HF_ = 9.7 Hz, H-3′,5′), 8.51 (2H, 2H, dd, *J*_HH_ *=* 8.7 Hz and *J*_HF_ *=* 5.4 Hz, H-2′,6′), 8.57 (1H, s H-8), 8.78 (1H, s, H-5); ^13^C-NMR (75 MHz, DMSO-*d*_6_) 20.4, 20.5, 20.6, 20.7, 20.8, 61.4, 68.3, 71.6, 71.7, 72.7, 98.8, 104.7, 105.1, 116.3 (d, ^2^*J*_CF_ = 21.8 Hz), 118.9, 119.7, 120.7, 130.4 (d, ^4^*J*_CF_ = 3.4 Hz), 130.9 (d, ^3^*J*_CF_ = 8.0 Hz), 156.0, 157.8, 164.1 (d, ^1^*J*_CF_ = 251.9 Hz), 169.5, 169.9, 170.0, 170.3, 171.7; HRMS (ES^+^): *m*/*z* [M + H]^+^ calc for C_29_H_26_O_12_F^35^ClI: 747.0142; found 747.0141.

*7-Chloro-2-(4-chlorophenyl)-6-iodo-4H-chromen-4-one-3-O-2,3,4,6-O-tetraacetyl-β-d-glucopyranoside* (**2g**): White solid (0.28 g, 69%), R*_f_* (50% EtOAc‒hexane) 0.63, m.p. 252–257 °C; ν_max_ (ATR) 482, 556, 761, 1023, 1160, 1208, 1422, 1639, 1747, 2651, 3063, 3075 cm^−1^; ^1^H-NMR (300 MHz, DMSO-*d*_6_) 1.90 (3H, s, -C(O)CH_3_), 1.99 (3H, s -C(O)CH_3_), 2.01 (3H, s, -C(O)CH_3_), 2.13, (3H, s, -C(O)CH_3_), 3.57–3.63 (1H, m, aliphatic), 3.93–3.98 (2H, m, aliphatic), 5.07 (1H, t, *J* = 9.6 Hz, aliphatic), 5.15–5.32 (2H, m, aliphatic), 5.57 (1H, d, *J* = 7.5 Hz, aliphatic), 7.47 (2H, d, *J* = 8.7 Hz, H-3′,5′), 7.69 (1H, s, H-8), 8.00 (2H, d, *J* = 9.0 Hz, H-2′,6′), 8.67 (1H, s, H-5); ^13^C-NMR (75 MHz, DMSO-*d*_6_) 20.5, 20.6, 20.7, 20.8, 61.1, 68.0, 71.4, 71.8, 72.5, 93.8, 98.9, 118.8, 123.8, 128.4, 128.6, 130.4, 136.5, 137.2, 137.5, 143.9, 154.8, 156.6, 169.4, 169.9, 170.0, 170.3, 171.9; HRMS (ES^+^): *m*/*z* [M + H]^+^ calc for C_29_H_26_O_12_^35^Cl_2_I: 762.9846; found 762.98426.

*7-Chloro-6-iodo-2-(4-methoxyphenyl)-4H-chromen-4-one-3-O-2,3,4,6-O-tetraacetyl-β-d-glucopyranoside* (**2h**): White solid (0.28 g, 78%), R*_f_* (50% EtOAc‒hexane) 0.71, m.p. 161–162 °C; ν_max_ (ATR) 471, 587, 760, 1027, 1161, 1203, 1418, 1632, 1750, 2654, 3070, 3071 cm^−1^; ^1^H-NMR (300 MHz, DMSO-*d*_6_) 1.88 (3H, s, -C(O)CH_3_), 2.02 (3H, s -C(O)CH_3_), 2.04 (3H, s, -C(O)CH_3_), 2.12 (3H, s, -C(O)CH_3_), 3.62–3.66 (1H, m, aliphatic), 3.90 (3H, s, -OCH_3_), 3.90–3.98 (2H, m, aliphatic), 5.00 (1H, t, *J* = 9.6 Hz, aliphatic), 5.18–5.32 (2H, m, aliphatic), 5.67 (1H, d, *J* = 7.5 Hz, aliphatic), 7.00 (2H, d, *J* = 9.0 Hz, H-3′,5′), 7.68 (1H, s, H-8), 8.01 (2H, d, *J* = 9.6 Hz, H-2′,6′), 8.65 (1H, s, H-5); ^13^C-NMR (75 MHz, DMSO-*d*_6_) 20.4, 20.5, 20.6, 20.7, 20.8, 55.4, 61.4, 68.3, 71.6, 71.7, 72.7, 93.3, 98.9, 113.7, 118.7, 122.2, 123.9, 131.0, 135.8, 137.1, 143.4, 154.8, 157.7, 161.9, 169.5, 170.0, 170.3, 171.7; HRMS (ES^+^): *m*/*z* [M + H]^+^ calc for C_30_H_29_O_13_^35^ClI: 759.0341; found 759.0338.

*7-Bromo-6-iodo-2-phenyl-4H-chromen-4-one-3-O-2,3,4,6-O-tetraacetyl-β-d-glucopyranoside* (**2i**): Brown solid (0.28 g, 80 %), R*_f_* (50% EtOAc‒hexane) 0.65, m.p.278–279 °C; ν_max_ (ATR) 468, 601, 691, 838, 917, 1033, 1191, 1219, 1422, 1591, 1643, 1738 cm^−1^; ^1^H-NMR (500 MHz, DMSO-*d*_6_) 1.81 (3H, s, -C(O)CH_3_), 1.94 (3H, s -C(O)CH_3_), 1.95 (3H, s, -C(O)CH_3_), 2.03 (3H, s, -C(O)CH_3_), 3.87–4.0 (3H, m, aliphatic), 4.89–5.00 (2H, m, aliphatic), 5.40 (1H, t, *J* = 9.0 Hz, aliphatic), 5.75–5.31 (2H, m, aliphatic), 7.50–7.56 (3H, m, H-4′ and H-3′,5′), 8.01 (2H, d, *J* = 7.5 Hz, H-2′,6′), 8.25 (1H, s H-8), 8.43 (1H, s, H-5); ^13^C-NMR (125 MHz, DMSO-*d*_6_) 20.7, 20.8, 20.9, 61.6, 68.3, 71.1, 71.8, 72.2, 98.5, 98.6, 123.3, 124.3, 128.7, 129.0, 129.5, 130.3, 131.7, 135.0, 136.0, 136.6, 154.7, 157.4, 169.7, 169.9, 170.1, 171.9; HRMS (ES^+^): *m*/*z* [M + H]^+^ calc for C_29_H_27_O_12_^79^BrI: 772.9731; found 772.9725.

*7-Bromo*-2-*(4-fluorophenyl)-6-iodo-4H-chromen-4-one-3-O-2,3,4,6-O-tetraacetyl-β-d-glucopyranoside* (**2j**): Brown solid (0.23 g, 72%), R*_f_* (50% EtOAc‒hexane) 0.69, m.p. 248–249 °C; ν_max_ (ATR) 475, 566, 770, 1021, 1160, 1218, 1433, 1631, 1758, 2644, 3060, 3075 cm^−1^; ^1^H-NMR (300 MHz, DMSO-*d*_6_) 1.17 (3H, s, -C(O)CH_3_), 2.00 (3H, s -C(O)CH_3_), 2.01 (3H, s, -C(O)CH_3_), 2.10 (3H, s, -C(O)CH_3_), 3.64 (1H, d, *J* = 9.3 Hz, aliphatic), 3.95 (2H, s, aliphatic), 5.07 (1H, t, *J* = 9.3 Hz, aliphatic), 5.15–5.31 (2H, m, aliphatic), 5.67 (1H, d, *J* = 7.8 Hz, aliphatic), 7.56 (2H, dd, *J*_HH_ *=* 8.7 and *J*_HF_ = 9.7 Hz, H-3′,5′), 8.10 (1H, s, H-8), 8.29 (2H, dd, *J*_HH_ *=* 8.7 Hz and *J*_HF_ *=* 5.4 Hz, H-2′,6′), 8.50 (1H, s, H-5); ^13^C-NMR (75 MHz, DMSO-*d*_6_) 20.4, 20.5, 20.6, 20.7, 20.8, 61.4, 68.2, 71.6, 71.7, 72.6, 77.8, 98.8, 104.7, 105.1, 116.2 (d, ^2^*J*_CF_ = 21.8 Hz), 118.8, 119.6, 120.5, 130.4 (d, ^4^*J*_CF_ = 3.5 Hz), 131.0 (d, ^3^*J*_CF_ = 8.0 Hz), 155.7, 155.9, 157.8, 164.1 (d, ^1^*J*_CF_ = 251.9 Hz), 169.9, 170.0, 170.3, 171.7; HRMS (ES^+^): *m*/*z* [M + H]^+^ calc for C_29_H_26_O_12_F^79^BrI: 790.9636; found 790.9631.

*7-Bromo-2-(4-chlorophenyl)-6-iodo-4H-chromen-4-one-3-O-2,3,4,6-O-tetraacetyl-β-d-glucopyranoside* (**2k**): Brown solid (0.25 g, 74%), R*_f_* (50% EtOAc‒hexane) 0.70, m.p. 298–299 °C; ν_max_ (ATR) 478, 570, 769, 1027, 1162, 1208, 1430, 1635, 1760, 2654, 3061, 3072 cm^−1^; ^1^H-NMR (500 MHz, DMSO-*d*_6_) 1.82 (3H, s, -C(O)CH_3_), 1.94 (6H, s, 2 x -C(O)CH_3_), 2.04 (3H, s, -C(O)CH_3_), 3.82–3.97 (3H, m, aliphatic), 4.90–5.03 (1H, m, aliphatic), 5.38 (1H, t, *J* = 10.0 Hz, aliphatic), 5.68 (1H, d, *J* = 8.0 Hz, aliphatic), 7.61 (2H, d, *J* = 9.0 Hz, H-3′,5′), 8.10 (2H, d, *J* = 8.5 Hz, H-2′,6′), 8.12 (1H, s H-8), 8.44 (1H, s, H-5); ^13^C-NMR (125 MHz, DMSO-*d*_6_) 20.6, 20.7, 20.8, 21.0, 61.5, 68.2, 71.1, 71.7, 72.1, 95.6, 99.0, 120.0, 124.0, 128.8, 129.1, 131.3, 136.3, 136.5, 136.7, 143.1, 155.0, 156.4, 169.7, 169.8, 169.9, 170.1, 171.7; HRMS (ES^+^): *m*/*z* [M + H]^+^ calc for C_29_H_26_O_12_^35^Cl^79^BrI: 806.9341; found 806.9338.

*7-Bromo-6-iodo-2-(4-methoxyphenyl)-4H-chromen-4-one-3-O-2,3,4,6-O-tetraacetyl-β-d-glucopyranoside* (**2l**): Brown solid (0.24 g, 74%), R*_f_* (50% EtOAc‒hexane) 0.64, m.p. 266–267 °C; ν_max_ (ATR) 508, 525, 589, 655, 838, 1027, 1182, 1216, 1426, 1890, 1641, 1737, 2360, 2921, 3078 cm^−1^; ^1^H-NMR (300 MHz, DMSO-*d*_6_) 1.88 (3H, s, -C(O)CH_3_), 2.00 (3H, s -C(O)CH_3_), 2.02 (3H, s, -C(O)CH_3_), 2.17 (3H, s, -C(O)CH_3_), 3.61-3.66 (1H, m, aliphatic), 3.90 (3H, s, -OCH_3_), 3.92–3.98 (2H, m, aliphatic), 5.08 (1H, t, *J* = 9.3 Hz, aliphatic), 5.18–5.23 (2H, m, aliphatic), 5.70 (1H, d, *J* = 7.5 Hz, aliphatic), 7.00 (2H, d, *J* = 9.0 Hz, H-3′,5′), 7.87 (1H, s, H-8), 8.05 (2H, d, *J* = 8.7 Hz, H-2′,6′), 8.64 (1H, s, H-5); ^13^C-NMR (75 MHz, DMSO-*d*_6_) 20.5, 20.6, 20.8, 55.4, 61.4, 68.2, 71.6, 71.7, 72.3, 96.3, 98.8, 113.7, 122.2, 124.3, 127.8, 131.0, 134.8, 136.9, 154.5, 157.6, 161.9, 169.5, 169.9, 170.0, 170.3, 171.9; HRMS (ES^+^): *m*/*z* [M + H]^+^ calc for C_30_H_29_O_13_^79^BrI: 802.9836; found 802.9832.

*6-Iodo-7-methoxy-2-phenyl-4H-chromen-4-one-3-O-2,3,4,6-O-tetraacetyl-β-d-glucopyranoside* (**2m**): Brown solid (0.29 g, 81 %), R*_f_* (50% EtOAc‒hexane) 0.62, m.p. 169–170 °C; ν_max_ (ATR) 510, 528, 579, 653, 840, 1024, 1172, 1213, 1422, 1590, 1644, 1731, 2360, 2918, 3070 cm^−1^; ^1^H (300 MHz, DMSO-*d*_6_) 1.80 (3H, s, -C(O)CH_3_), 1.94 (3H, s -C(O)CH_3_), 1.96 (3H, s, -C(O)CH_3_), 2.04 (3H, s, -C(O)CH_3_), 3.87–4.01 (3H, m, H-3′,5′ and H-4′), 3.95 (3H, s, -OCH_3_), 4.86–5.00 (2H, m, aliphatic), 5.39 (1H, t, *J* = 9.9 Hz, aliphatic), 5.80 (1H, d, *J* = 8.1 Hz, aliphatic), 7.33 (1H, s H-8), 7.53–7.55 (3H, m), 8.06 (2H, dd, *J* = 2.4 Hz and 9.3 Hz, H-2′,6′), 8.32 (1H, s, H-5); ^13^C (75 MHz, DMSO-*d*_6_) 20.7, 20.8, 21.0, 57.9, 61.6, 68.3, 71.0, 71.7, 72.1, 84.9, 98.5, 100.7, 119.1, 128.7, 129.3, 130.6, 131.4, 135.2, 136.1, 156.7, 157.0, 162.2, 162.7, 169.8, 169.9, 170.1, 171.6; HRMS (ES^+^): *m*/*z* [M + H]^+^ calc for C_30_H_30_O_13_I: 725.0731; found 725.0726.

*2-(4-Fluorophenyl)-6-Iodo-7-methoxy-4H-chromen-4-one-3-O-2,3,4,6-O-tetraacetyl-β-d-glucopyranoside* (**2n**): Brown solid (0.29 g, 79 %), R*_f_* (50% EtOAc‒hexane) 0.65, m.p. 177–178 °C; ν_max_ (ATR) 512, 525, 569, 650, 837, 1027, 1168, 1212, 1426, 1596, 1642, 1727, 2361, 2915, 3072 cm^−1^; ^1^H-NMR (300 MHz, DMSO-*d*_6_) 1.87 (3H, s, -C(O)CH_3_), 1.99 (3H, s -C(O)CH_3_), 2.01 (3H, s, -C(O)CH_3_), 2.10 (3H, s, -C(O)CH_3_), 3.62 (1H, d, *J* = 9.3 Hz, aliphatic), 3.89 (3H, s, -OCH_3_) 3.95–3.97 (2H, m, aliphatic), 5.07 (1H, t, *J* = 8.1 Hz, aliphatic), 5.17–5.31 (2H, m, aliphatic), 5.56 (1H, d, *J* = 7.8 Hz, aliphatic), 7.00 (2H, *J*_HH_ *=* 8.7 and *J*_HF_ = 9.7 Hz, H-3′,5′′), 7.20 (1H, d, *J* = 7.5 Hz, H-8), 8.00 (2H, dd, *J*_HH_ *=* 8.7 Hz and *J*_HF_ *=* 5.4 Hz, H-2′,6′), 8.61 (1H, d, *J* = 7.2 Hz, H-5); ^13^C-NMR (75 MHz, DMSO-*d*_6_) 20.5, 20.6, 20.8, 55.4, 61.4, 68.2, 71.6, 71.7, 72.7, 77.8, 98.9, 104.8, 105.1, 116.4 (d, ^2^*J*_CF_ = 21.8 Hz), 118.8, 119.7, 120.6, 130.5 (d, ^4^*J*_CF_ = 3.5 Hz), 130.9 (d, ^3^*J*_CF_ = 8.0 Hz), 155.8, 156.0, 157.8, 164.1 (d, ^1^*J*_CF_ = 251.9 Hz), 169.5, 169.9, 170.1, 170.3, 171.7; HRMS (ES^+^): *m*/*z* [M + H]^+^ calc for C_30_H_29_O_13_FI: 743.0637; found 743.0635.

*2-(4-Chlorophenyl)-6-iodo-7-methoxy-4H-chromen-4-one-3-O-2,3,4,6-O-tetraacetyl-β-d-glucopyranoside* (**2o**): Brown solid (0.27 g, 78%), R*_f_* (50% EtOAc‒hexane) 0.63, m.p. 184–185 °C; ν_max_ (ATR) 488, 579, 770, 1030, 1164, 1200, 1420, 1633, 1750, 2651, 3070, 3071 cm^−1^; ^1^H-NMR (500 MHz, DMSO-*d*_6_) 1.80 (3H, s, -C(O)CH_3_), 1.95 (3H, s -C(O)CH_3_), 1.96 (3H, s, -C(O)CH_3_), 2.04 (3H, s, -C(O)CH_3_), 3.82–4.00 (3H, m, aliphatic), 3.84 (3H, s, -OCH_3_), 4.93–5.04 (2H, m, aliphatic), 5.39 (1H, t, *J* = 9.5 Hz, aliphatic), 5.76 (1H, d, *J* = 7.5 Hz, aliphatic), 7.01 (2H, d, *J* = 9.0 Hz, H-3′,5′), 8.01 (2H, d, *J* = 7.0 Hz, H-2′,6′), 8.10 (1H, s H-8), 8.43 (1H, s, H-5); ^13^C-NMR (125 MHz, DMSO-*d*_6_) 20.7, 20.8, 20.9, 57.9, 61.6, 68.3, 71.0, 71.8, 72.1, 84.9, 98.5, 100.7, 119.1, 128.1, 128.9, 130.4, 131.3, 134.2, 135.8, 156.7, 157.0, 162.2, 162.8, 169.8, 169.9, 170.1, 171.6; HRMS (ES^+^): *m*/*z* [M + H]^+^ calc for C_30_H_29_O_13_^35^ClI: 759.0341; found 759.0336.

*6-Iodo-7-methoxy-2-(4-methoxyphenyl)-4H-chromen-4-one-3-O-2,3,4,6-O-tetraacetyl-β-d-glucopyranoside* (**2p**): Brown solid (0.30 g, 83%), R*_f_* (50% EtOAc‒hexane) 0.59, m.p. 129–130 °C; ν_max_ (ATR) 482, 568, 777, 1029, 1151, 1213, 1416, 1630, 1753, 2641, 3068, 3072 cm^−1^; ^1^H-NMR (500 MHz, DMSO-*d*_6_) 1.79 (3H, s, -C(O)CH_3_), 2.02 (3H, s -C(O)CH_3_), 1.95 (3H, s, -C(O)CH_3_), 1.96 (3H, s, -C(O)CH_3_), 3.84 (3H, s, -OCH_3_), 3.86–3.94 (3H, m, aliphatic), 3.97 (3H, s, -OCH_3_), 4.90–5.36 (2H, m, aliphatic), 5.38 (1H, t, *J* = 9.5 Hz, aliphatic), 5.77 (1H, d, *J* = 8.5 Hz, aliphatic), 7.10 (2H, d, *J* = 9.0 Hz, H-3′,5′), 7.36 (1H, s H-8), 8.01 (2H, d, *J* = 8.5 Hz, H-2′,6′), 8.35 (1H, s, H-5); ^13^C-NMR (125 MHz, DMSO-*d*_6_) 25.4, 25.5, 25.7, 39.6, 60.6, 62.7, 66.3, 70.1, 73.1, 75.0, 75.7, 76.7, 76.9, 89.3, 103.3, 105.5, 118.9, 119.1, 123.9, 127.5, 135.9, 140.1, 161.4, 161.7, 166.5, 166.9, 174.5, 174.9, 174.8, 176.2; HRMS (ES^+^): *m*/*z* [M + H]^+^ calc for C31H32O_14_I: 755.0837; found 755.0833.

### 3.3. In Vitro Cholinesterase (AChE and BChE) Inhibition Assays

Compounds **2a**–**2l** were evaluated for anticholinesterase activities by the Ellman’s method [36] with slight modification as described in previous investigations [22,37,38]. The absorbances were determined spectrometrically at 412 nm on a Varioskan flash spectrophotometer (Thermo Scientific, Waltham, MA, USA). The IC_50_ and standard deviation values were determined graphically using Graph Pad Prism.

### 3.4. In Vitro β-Secretase Inhibitory Assays

The inhibitory properties of compounds **2a**–**2l** on β-secretase were evaluated by a fluorescence resonance energy transfer (FRET) assay (Pan Vera) with a recombinant baculovirus-expressed β-secretase and a specific substrate (Rh-EVNLDAEFK-Quencher) according to manufacturer instructions as described in our previous study [22]. The inhibition ratio was calculated using the following equation:

Inhibition (%) = [1 − (S − S_0_)/(C − C_0_)] × 100 (1)

where C is the fluorescence of control (enzyme, assay buffer, and substrate) after 60 min of incubation, C_0_ is the fluorescence of control at time 0, S is the fluorescence of tested samples (enzyme, sample solution, and substrate) after 60 min of incubation, and S_0_ the fluorescence of the tested samples at time 0.

### 3.5. Kinetic Studies of ***2l*** and ***2p*** against ChEs and β-Secretase

Kinetic evaluation of **2l** and **2p** against ChEs or β-secretase were conducted following similar procedures previously employed for the corresponding flavonol precursors [22]. Compounds **2l** and **2p** were selected for the kinetic studies with substrate concentrations ranging from (0.1, 0.5, 2. 5, and 5µM) and (150, 300 and 450 nM) for ChEs or β-secretase, respectively. The Lineweaver–Burk plots (plots of the inverse of velocity (1/v) against the inverse of the substrate concentration (1/[S])) were used to ascertain the mode of inhibition of these compounds (**2l** and **2p**). On the other hand, the plots of 1/v against concentration of inhibitor at each concentration of substrate (the Dixon plot) were used to determine their inhibitor constants (Ki).

### 3.6. 2,2-Diphenyl-1-picrylhydrazyl (DPPH) Radical Scavenging Activity Assay

DPPH radical scavenging activity of compounds **2e**, **2g**, **2i**, **2k**, **2l**, **2n, and 2p** was evaluated following the literature method [39]. The compounds were evaluated against the natural antioxidants ascorbic acid (Sigma Aldrich, Saint Louis, MO, USA) and quercetin (Sigma Aldrich, Saint Louis, MO, USA) as positive controls. The test compounds at various concentration ranging from (0 to 40 µM) in DMSO were mixed with a solution of DPPH (0.20 mM) in methanol. The mixtures were incubated in the dark for 45 min and the absorbances were recorded at 512 nm [40,41]. All tests and analyses were run in triplicate and averaged. The inhibition was calculated in terms of percentage using the formula below:

DPPH radical scavenged (%) = [(AbC ‒ AbS)/AbC] × 100(2)

where AbC is absorbance of control and AbS the absorbance of the test sample. A graph of percentage inhibition of free-radical activity was plotted against concentration of the sample and the IC_50_ (compound concentration required to reduce the absorbance of the DPPH control solution by 50%) was obtained from the graph.

### 3.7. Molecular Docking

The CDOCKER module of Discovery Studio software (version 17.1.0.16143, Accelrys, San Diego, CA, USA) was used to investigate interactions of the title compounds with AChE, BChE and β-secretase. PDB structures used were as follows: for AChE was 1GQR; for BChE was 1P0I and for β-Secretase was 3IXJ. The proteins structures were prepared prior to docking using default settings of Discovery Studio except that co-crystalized ligands were removed. The compounds were drawn in Discovery Studio then prepared using default parameters prior to docking. The binding sites used to dock compounds represented co-crystalized ligand or substrate locations as identified by Discovery Studio software, the grid box centered at the geometrical center of the region of the co-crystallized ligand binding sphere. In instances where docking failed for the default sphere size, the radius of the docking sphere increased without affecting the grid box center and docking conducted to achieve results. The sphere size increased from 11.1 to 11.8 for 1GQR; from 12 to 14.2 for P0I and from 15.3 to 16.5 for 3IXJ. The best scoring pose without unfavorable interactions was selected and represented as 2D plots using Discovery Studio.

## 4. Conclusions

Compounds **2l** and **2p**, which have potential to exhibit dual inhibitory effect against AChE and BChE activities, were found to exhibit moderate inhibitory activity against β-secretase. Molecular docking studies of these compounds into AChE, BChE, and β-secretase binding sites predicted electrostatic and hydrophobic interactions to be the key factors that stabilize the enzyme–ligand complexes. The observed non-competitive inhibitory mode of compounds **2l** and **2p** confirmed by the kinetic studies against AChE activity indicates that the predicted interactions of these compounds with protein residues in the CAS and PAS do not exclude substrate binding. Docking of compounds **2l** and **2p** into β-secretase, on the other hand, showed no interaction with the catalytic aspartic acids Asp32 and Asp228. This prediction is consistent with the kinetic results indicating binding to the enzyme at sites affecting allosteric changes that decrease activity rather than competing with the substrate to bind the active site. The observed anticholinesterase activity and moderate free-radical scavenging potential of the *O*-glucopyranoside derivative **2p** demonstrate the significance of the liphophilic methoxy group on both ring-A and ring-B. Since methoxy-substituted flavonols and their glycosides are widely distributed in natural foods, these results provide important information for the evaluation of the role of a flavonoid-rich diet for the prevention of AD.

## Figures and Tables

**Table 1:** Substitution pattern for compounds **2a**–**2p.**

R	Designation of X for 2a–2p			
H	F (**2a**)	Cl (**2e**)	Br (**2i**)	OCH_3_ (**2m**)
F	F (**2b**)	Cl (**2f**)	Br (**2j**)	OCH_3_ (**2n**)
Cl	F (**2c**)	Cl (**2g**)	Br (**2k**)	OCH_3_ (**2o**)
OCH_3_	F (**2d**)	Cl (**2h**)	Br (**2l**)	OCH_3_ (**2p**)

**Table 2:** AChE and BChE inhibitory activities of flavonols **2a**–**2p**.

Compound	IC_50_ (µM)		SI	
	AChE	BChE	BChE/AChE	AChE/BChE
**2a**	18.72 ± 0.03	26.17 ± 0.01	1.40	0.71
**2b**	28.2 ± 0.02	21.9 ± 0.04	0.78	1.3
**2c**	24.74 ± 0.03	30.84 ± 0.04	1.25	0.80
**2d**	60.40 ± 0.05	19.25 ± 0.11	0.32	3.13
**2e**	34.44 ± 0.03	9.03 ± 0.12	0.26	3.85
**2f**	70.66 ± 0.01	31.09 ± 0.04	0.44	2.27
**2g**	31.44 ± 0.13	7.15 ± 0.01	0.23	4.35
**2h**	25.92 ± 0.02	10.10 ± 0.06	0.39	2.56
**2i**	70.56 ± 0.03	9.79 ± 0.04	0.14	7.14
**2j**	31.20 ± 0.05	40.93 ± 0.04	1.31	0.76
**2k**	5.18 ± 0.01	29.95 ± 0.02	5.78	0.17
**2l**	5.05 ± 0.02	7.24 ± 0.03	1.43	0.70
**2m**	56.21 ± 0.01	29.80 ± 0.06	0.53	1.89
**2n**	6.23 ± 0.05	34.21 ± 0.02	5.49	0.18
**2o**	28.12 ± 0.06	36.10 ± 0.02	1.28	0.78
**2p**	9.19 ± 0.03	5.21 ± 0.03	0.57	1.75
Donepezil	4.82 ± 0.01	6.01 ± 0.02	1.25	0.80

The values are given as mean ± standard deviation (SD) in three independent experiments. Selectivity Index (SI): the AChE selectivity index is defined as IC_50_ (BChE)/IC_50_ (AChE) and that of BChE as IC_50_ (AChE)/IC_50_ (BChE) affinity ratios.

**Table 3:** Inhibitory activity of compounds **2l** and **2p**.

Compounds	IC_50_ (µM)
**2l**	18.3 ± 0.01
**2p**	25.2 ± 0.15
Quercetin	13.7 ± 0.02

**Table 4:** Scavenging Activity of Compounds **2e**, **2g**, **2i**, **2k**, **2l**, **2n, and 2p**.

Compounds	IC_50_ (µM)
**2g**	13.1 ± 0.01
**2k**	11.4 ± 0.02
**2l**	21.6 ± 0.03
**2n**	14.4 ± 0.02
**2p**	17.9 ± 0.04
Ascorbic acid Quercetin	5.5 ± 0.02 3.1 ± 0.01

Each value represents mean ± SD; IC_50_: 50% effective concentration.

## Supplementary Materials

The following are available online. Copies of 1H- and 13C-NMR spectra of compounds **2a**–**2p** (Figure S1); and the Lineweaver–Burk and Dixon plots for **2l** and **2p** against AChE and BChE (Figure S2) and against β-secretase (Figure S3).
