# Supplementary material for: Inhibitory Effects of Novel 7-Substituted 6-iodo-3-O-Flavonol Glycosides against Cholinesterases and β-secretase Activities, and Evaluation for Potential Antioxidant Properties
Source: Molecules. 2019 Sep 26;24(19):3500. doi: 10.3390/molecules24193500 (PMC6804162; doi:10.3390/molecules24193500)
Supplement: Supplementary file 1 [file molecules-24-03500-s001.pdf]

Supplementary Material

Figure S1: Copies of ^{1}H- and ^{13}C-NMR spectra of compounds **2a–2p**.

Figure S2: Lineweaver-Burk and Dixon plots for compounds **2l** and **2p** against AChE and BChE.

Figure S2: Lineweaver-Burk and Dixon plots for compounds **2l** and **2p** against β-secretase.

Figure S1: **Copies of ¹H- and ¹³C-NMR spectra of compounds 2a–2p.**

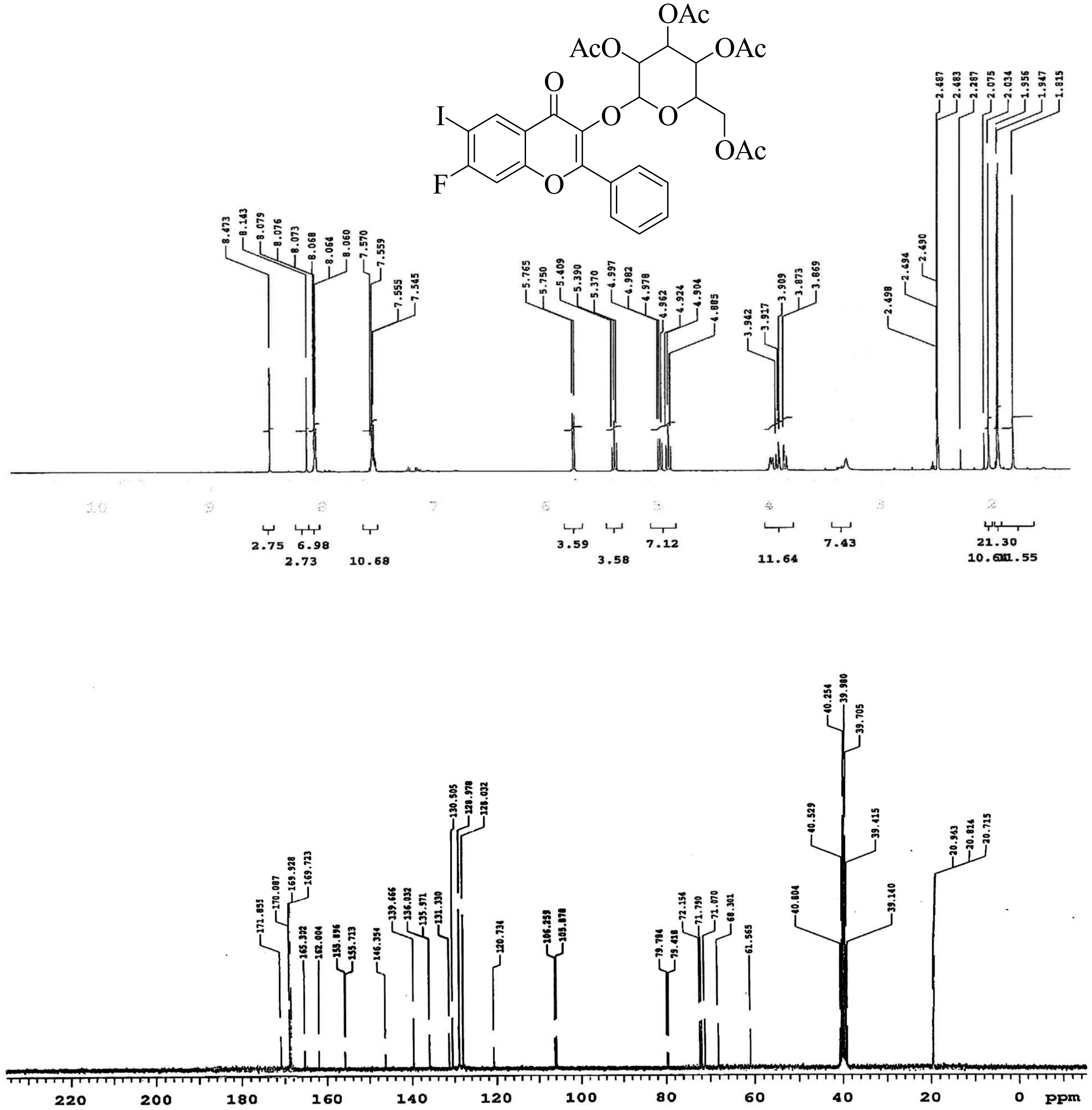

Figure S1.1: ¹H- and ¹³C-NMR spectra of **2a** in DMSO-d_6 at 500 and 125 MHz, respectively.

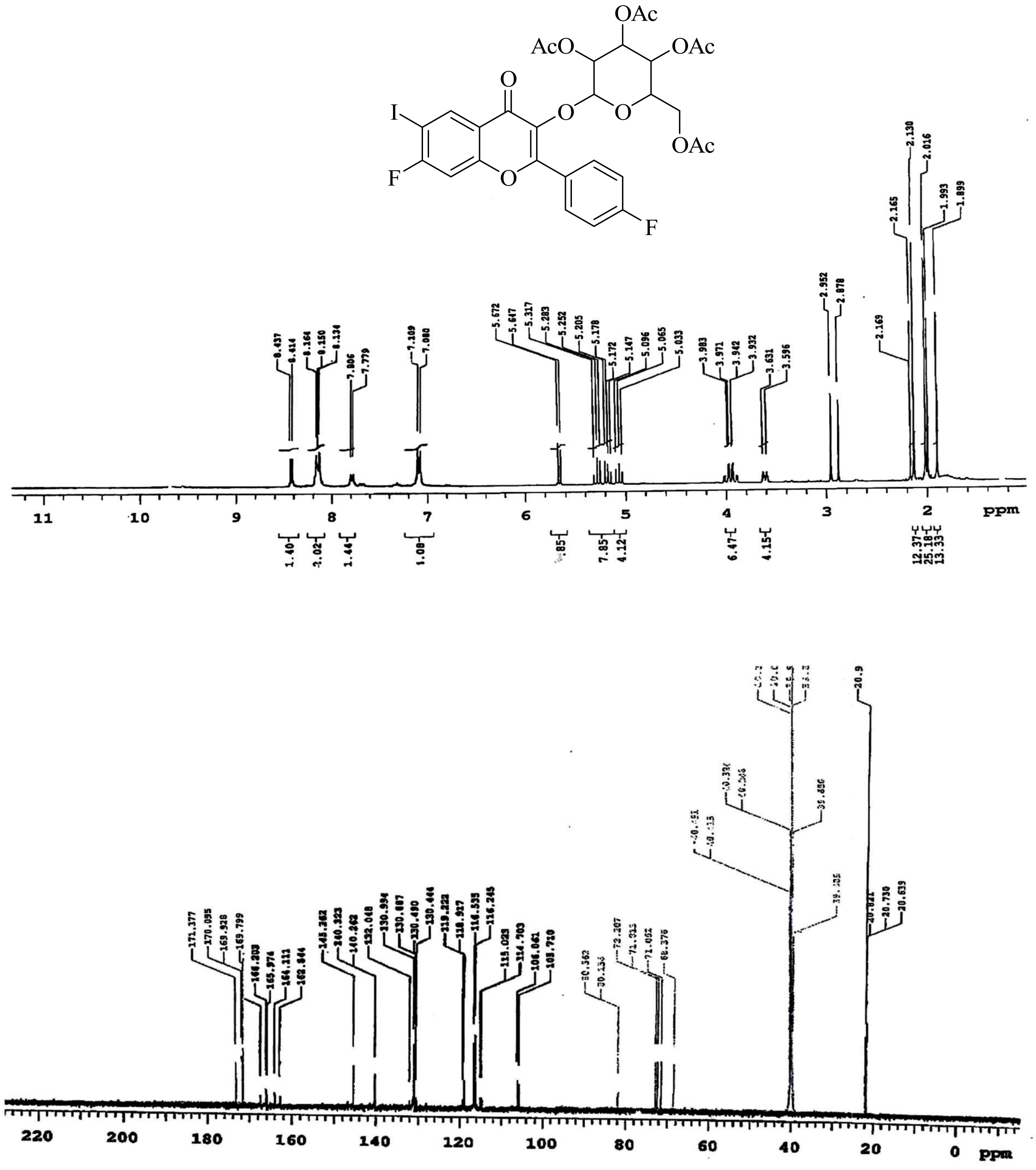

Figure S1.2: ^{1}H- and ^{13}C-NMR spectra of **2b** in DMSO-d_6 at 300 and 75 MHz, respectively.

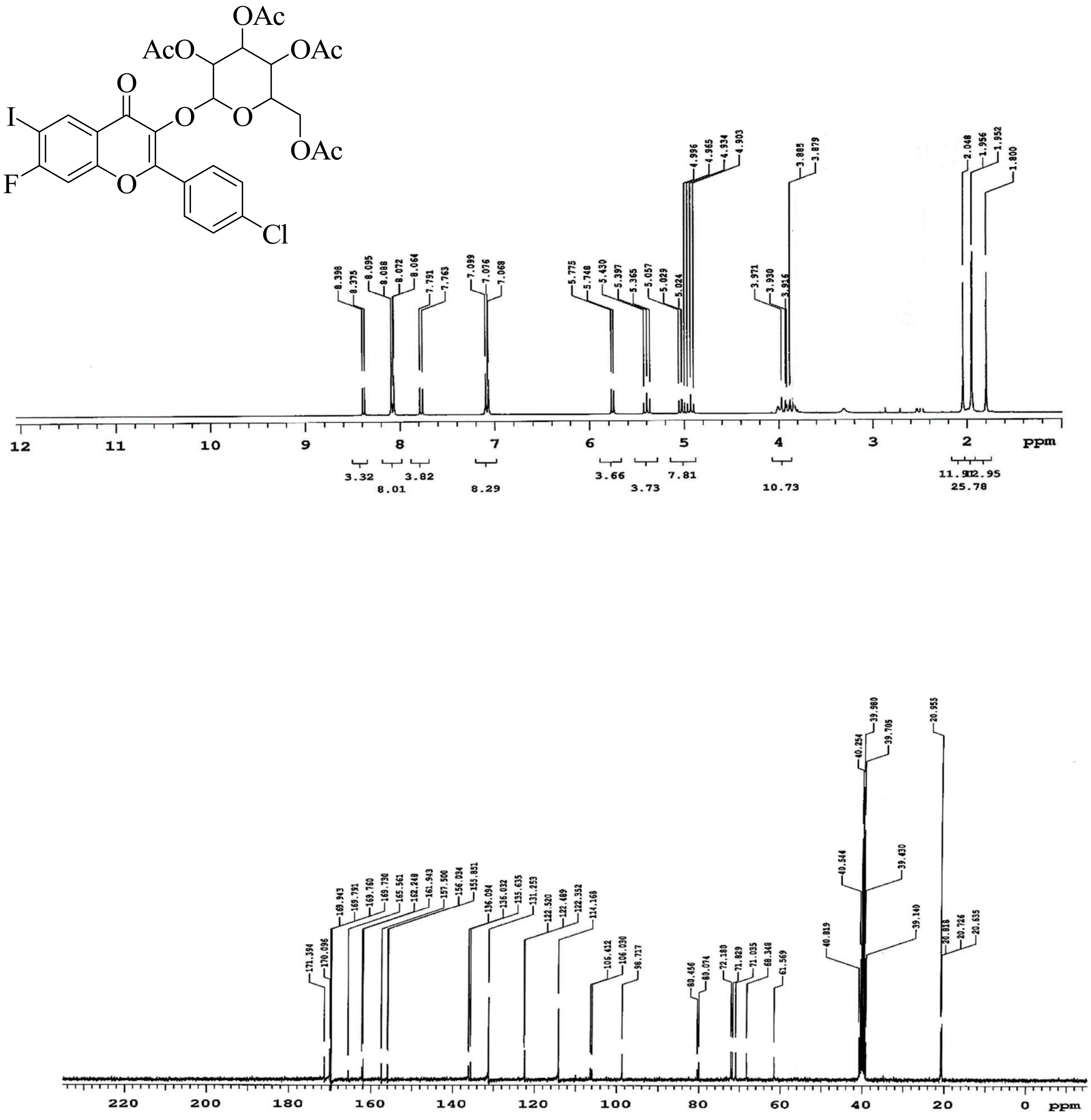

Figure S1.3: ^{1}H- and ^{13}C-NMR spectra of **2c** in DMSO-d_6 at 300 and 75 MHz, respectively.

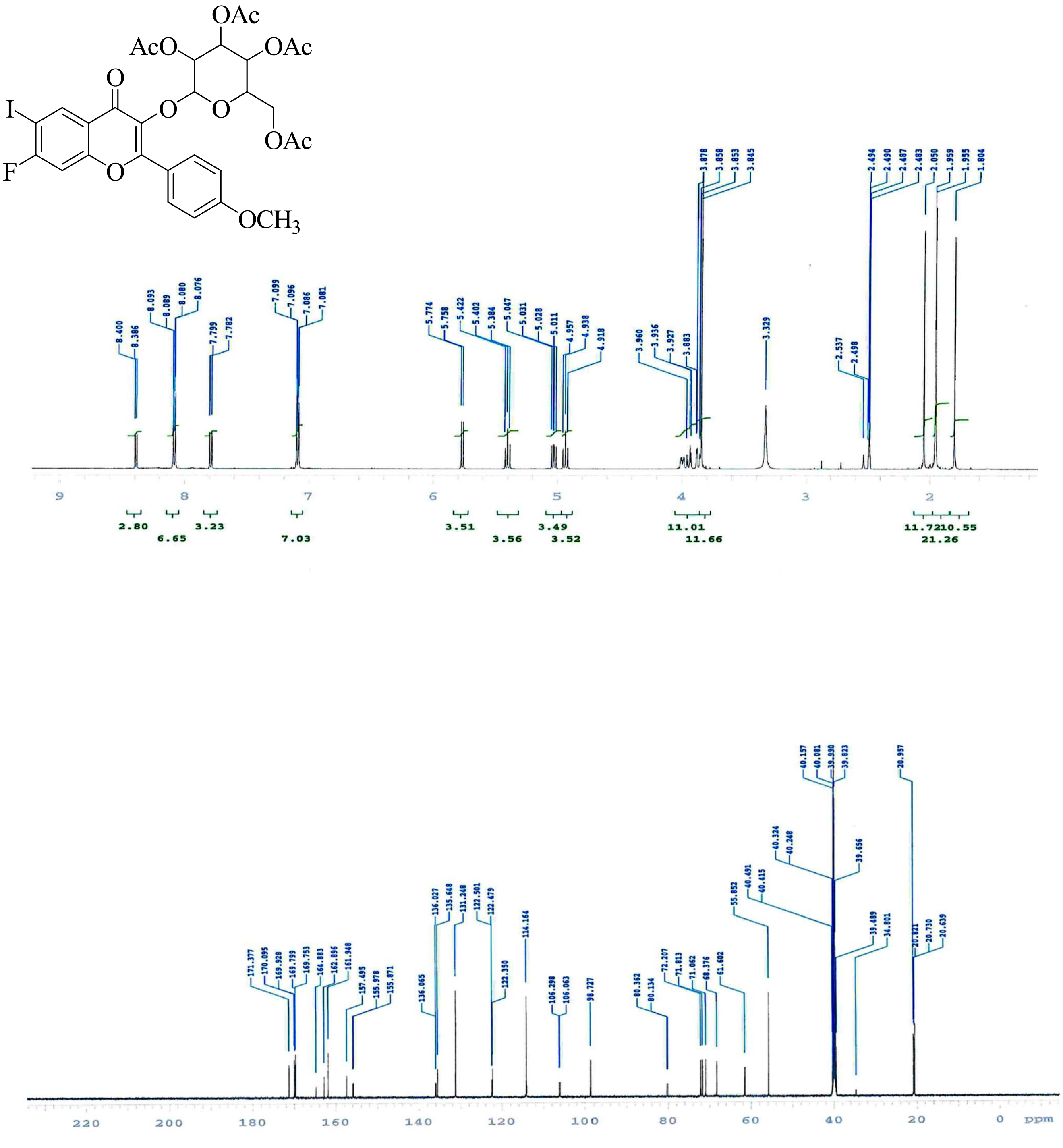

Figure S1.4: ^{1}H- and ^{13}C-NMR spectra of **2d** in DMSO-d_6 at 500 and 125 MHz, respectively.

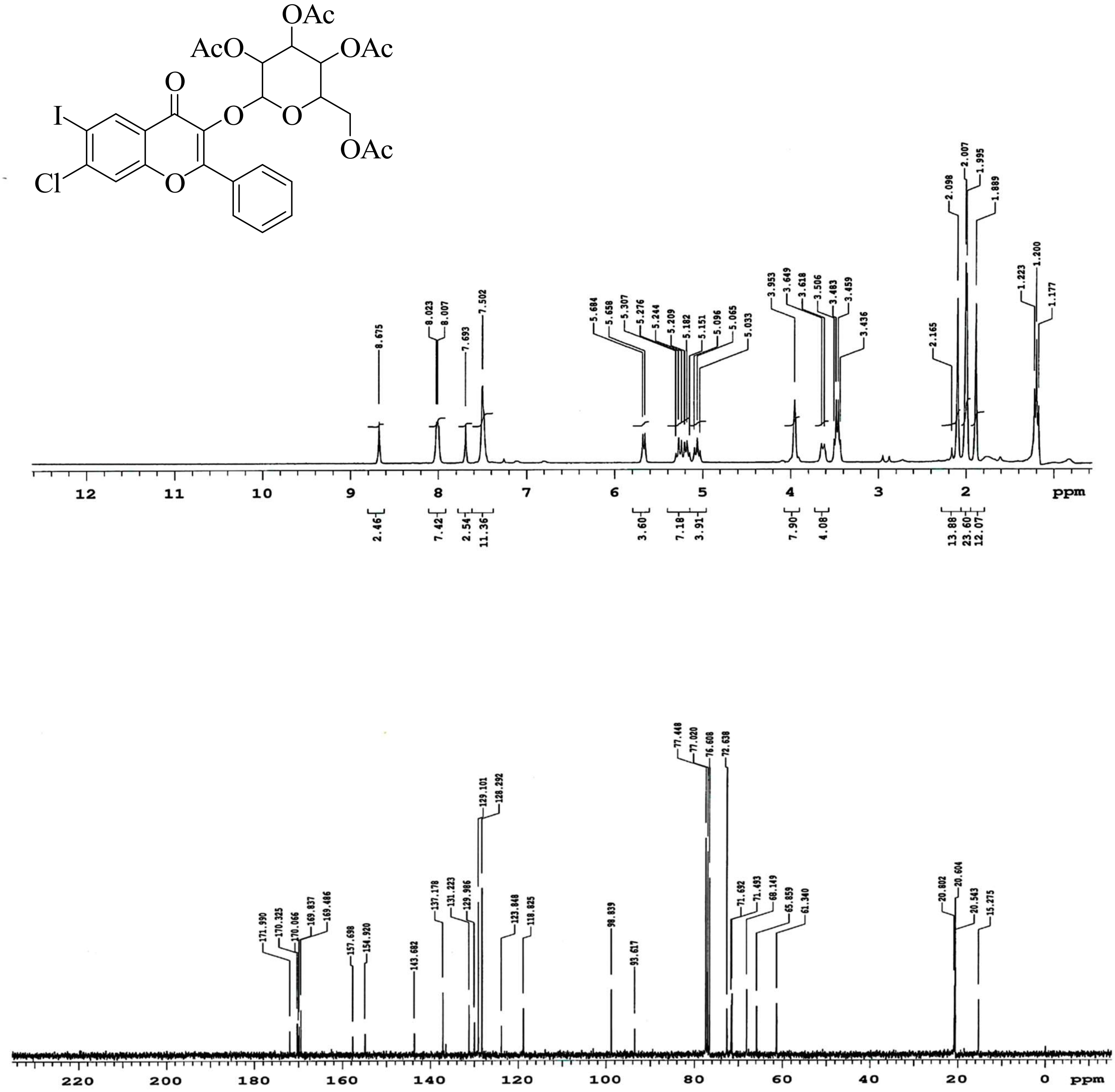

Figure S1.5: ^{1}H- and ^{13}C-NMR spectra of **2e** in $CDCl_3$ at 300 and 75 MHz, respectively.

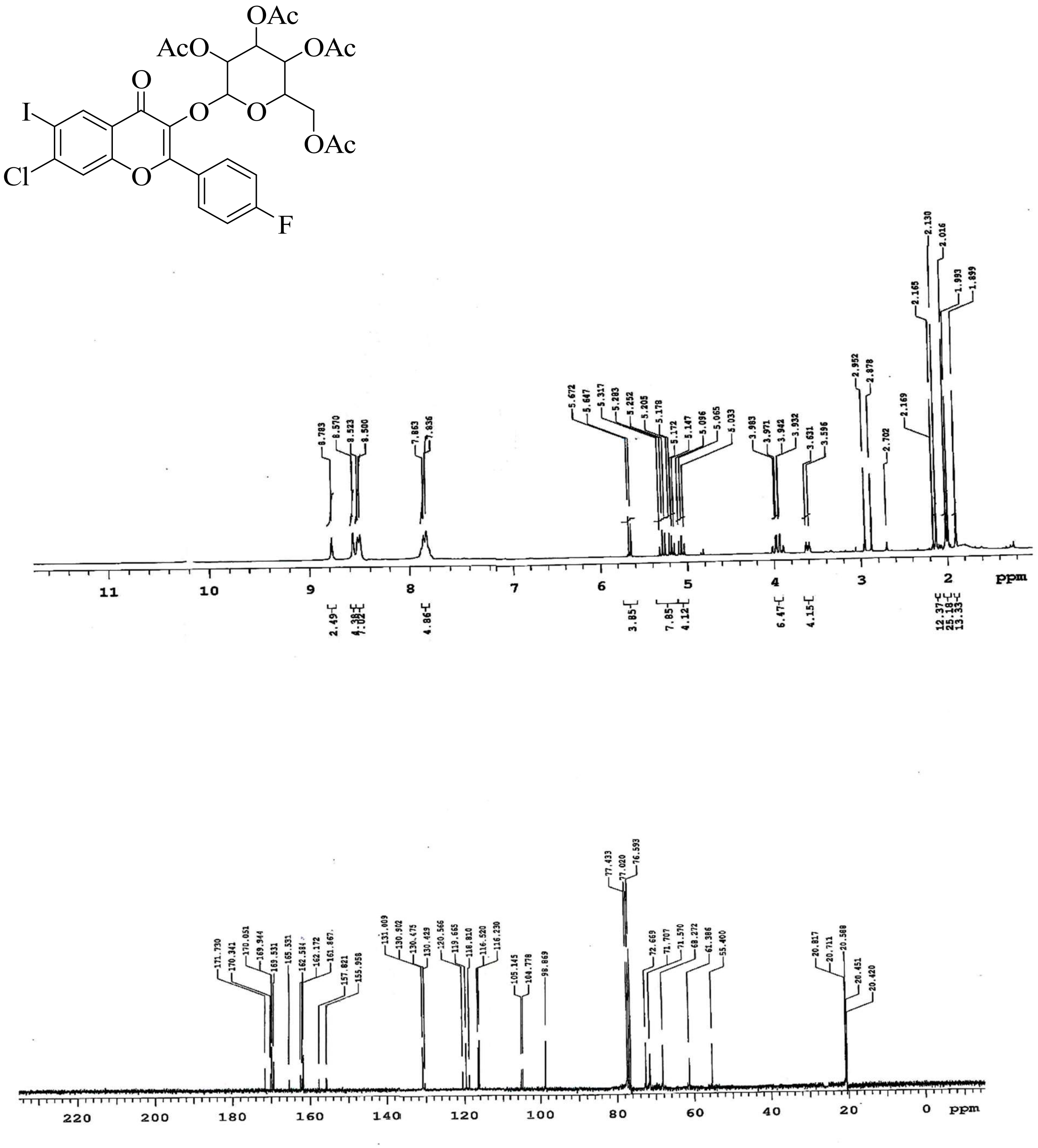

Figure S1.6: ^{1}H- and ^{13}C-NMR spectra of **2f** in $CDCl_3$ at 300 and 75 MHz, respectively.

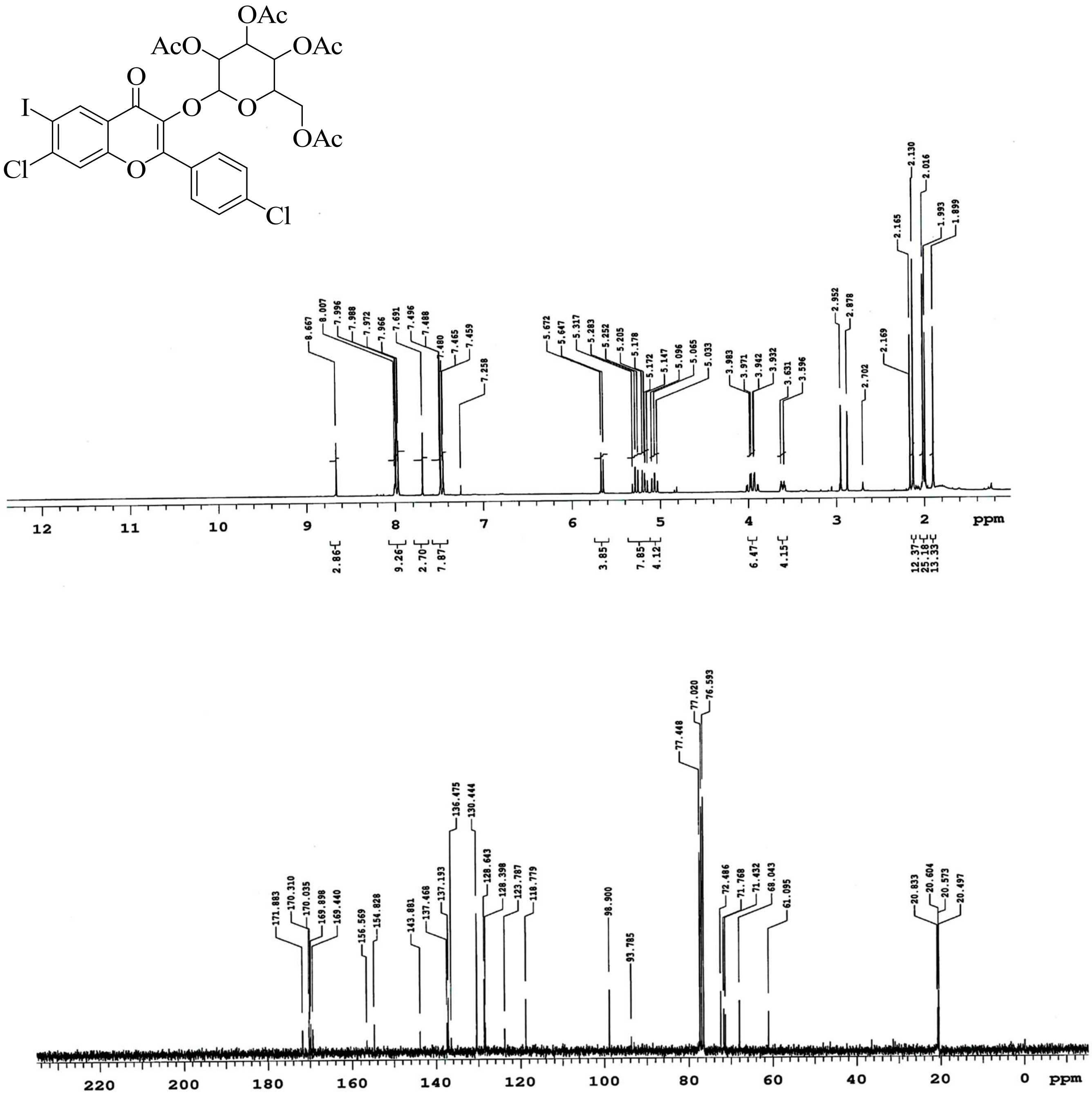

Figure S1.7: ^{1}H- and ^{13}C-NMR spectra of **2g** in $CDCl_3$ at 300 and 75 MHz, respectively.

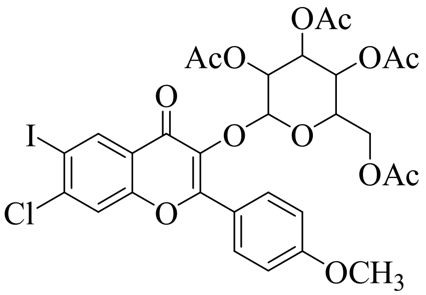

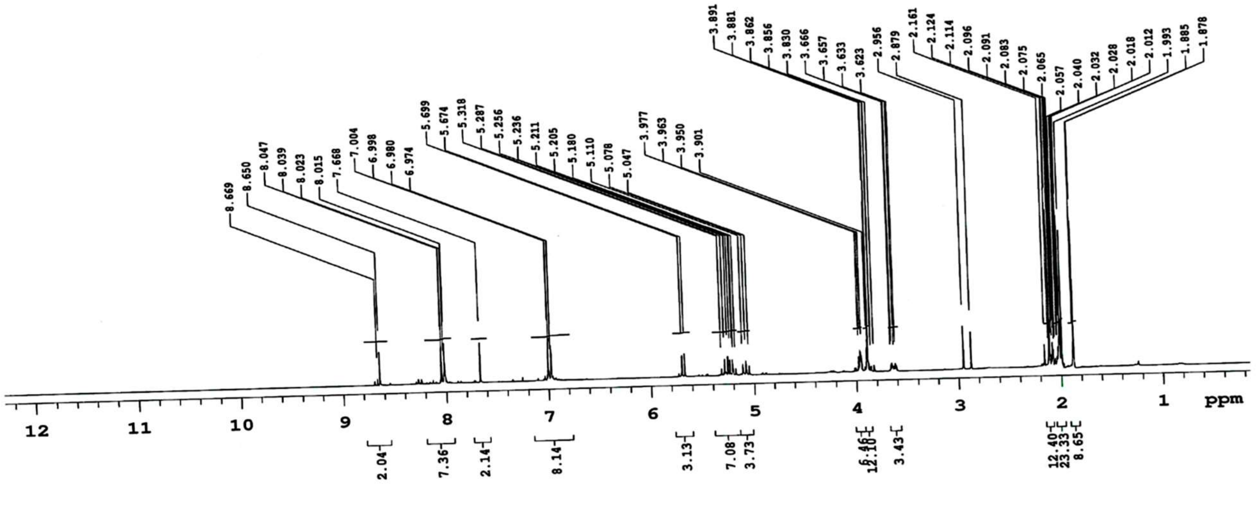

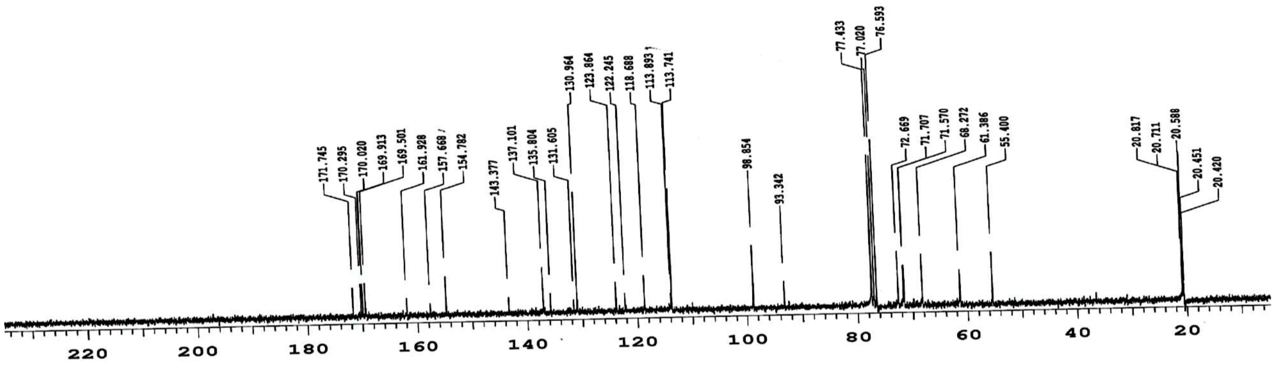

Figure S1.8: ^{1}H- and ^{13}C-NMR spectra of **2h** in $CDCl_3$ at 300 and 75 MHz, respectively.

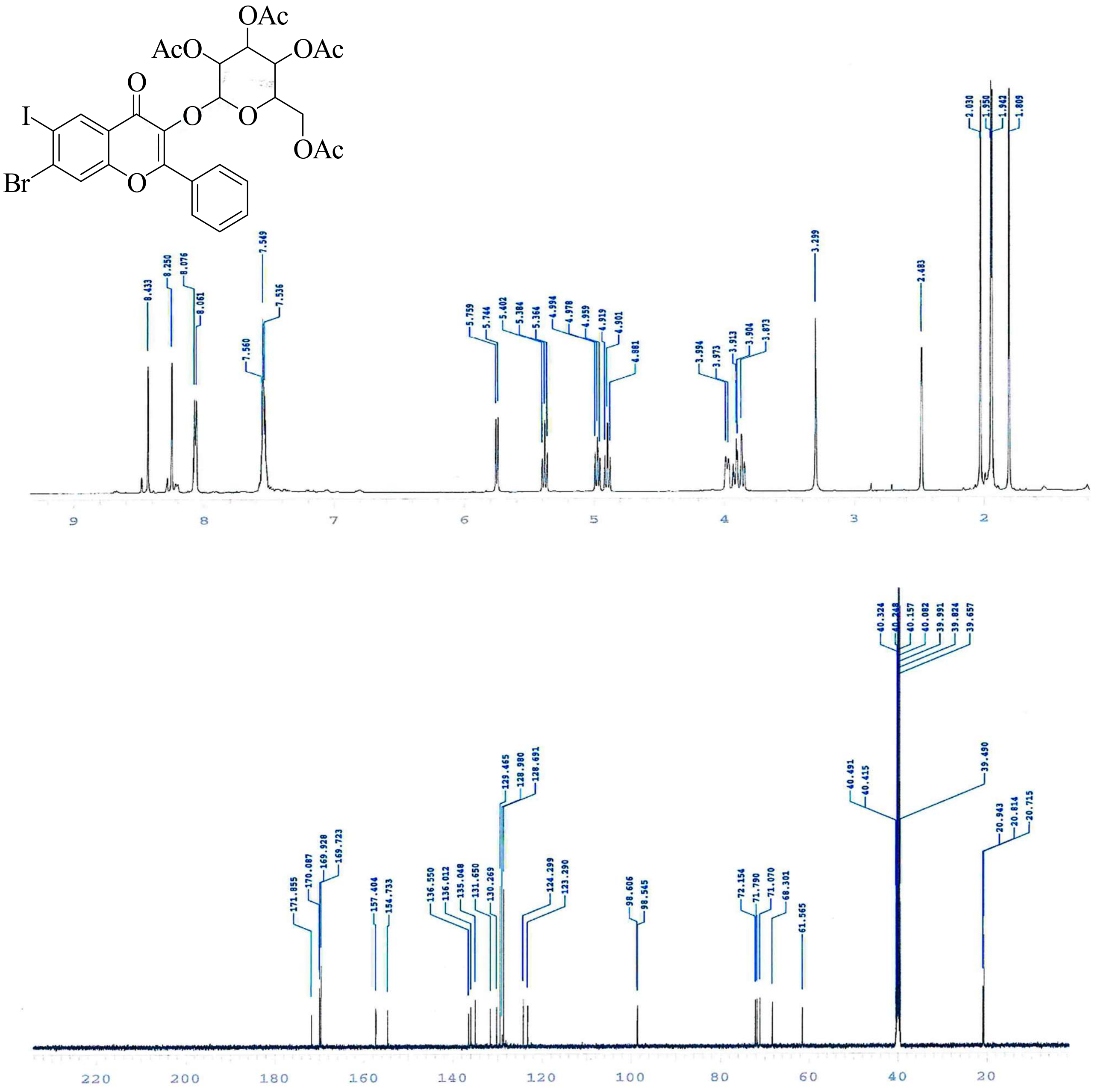

Figure S1.9: ^{1}H- and ^{13}C-NMR spectra of **2i** in DMSO-d_6 at 500 and 125 MHz, respectively.

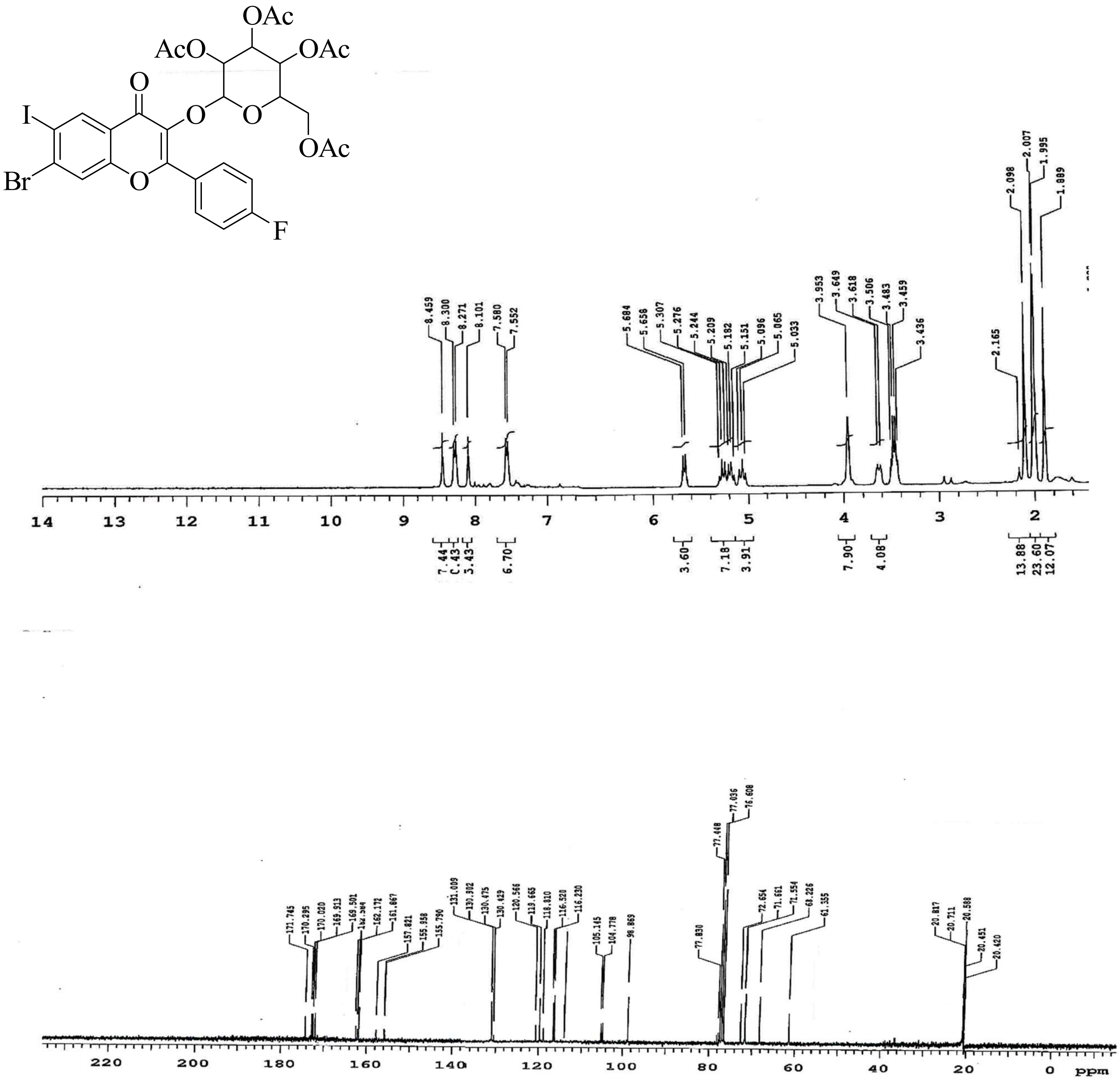

Figure S1.10: ^{1}H- and ^{13}C-NMR spectra of **2j** in $CDCl_3$ at 300 and 75 MHz, respectively.

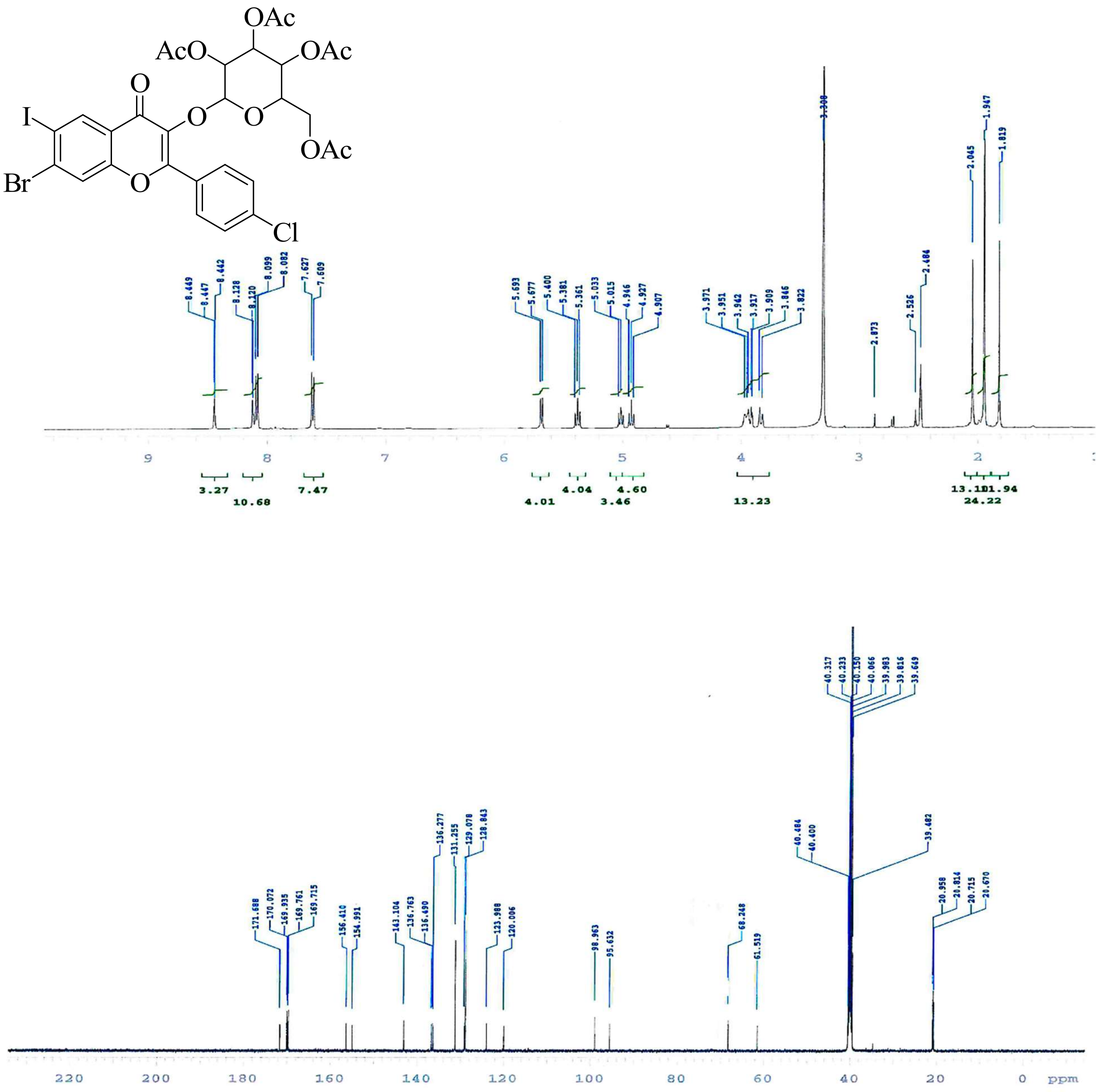

Figure S1.11: ^{1}H- and ^{13}C-NMR spectra of **2k** in DMSO-d_6 at 500 and 125 MHz, respectively.

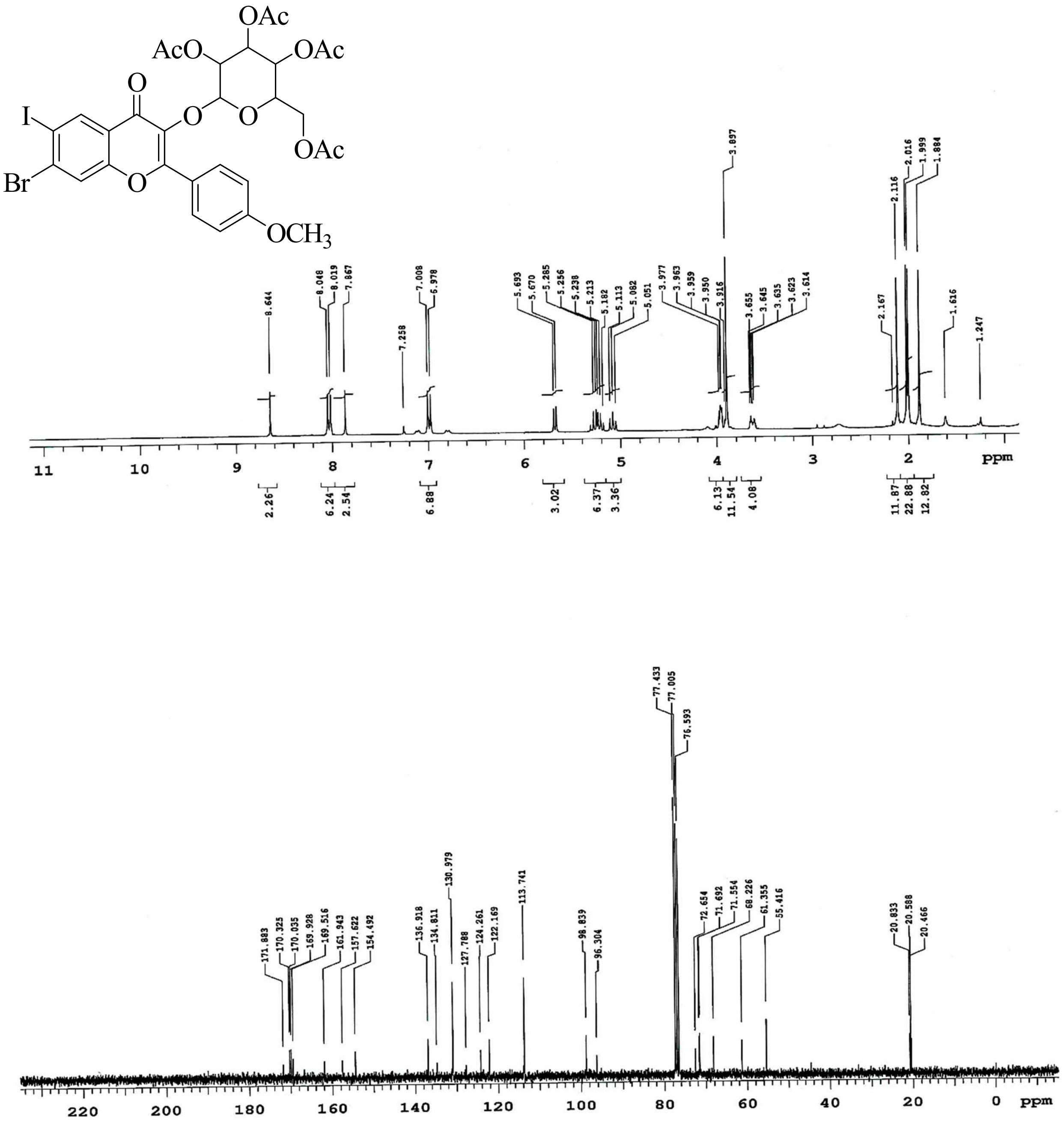

Figure S1.12: ^{1}H- and ^{13}C-NMR spectra of **2l** in $CDCl_3$ at 300 and 75 MHz, respectively.

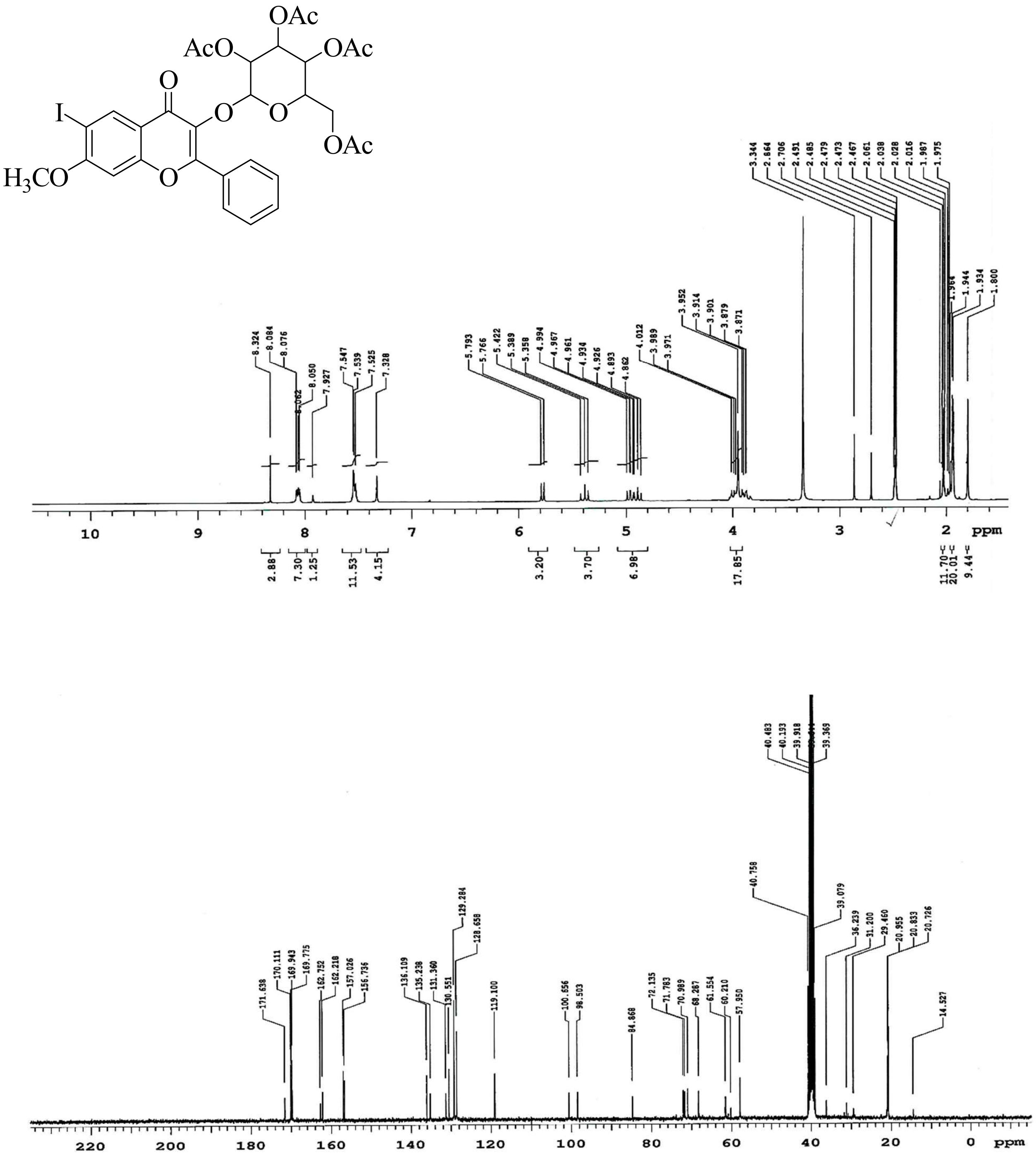

Figure S1.13: ^{1}H- and ^{13}C-NMR spectra of **2m** in DMSO-d_6 at 300 and 75 MHz, respectively

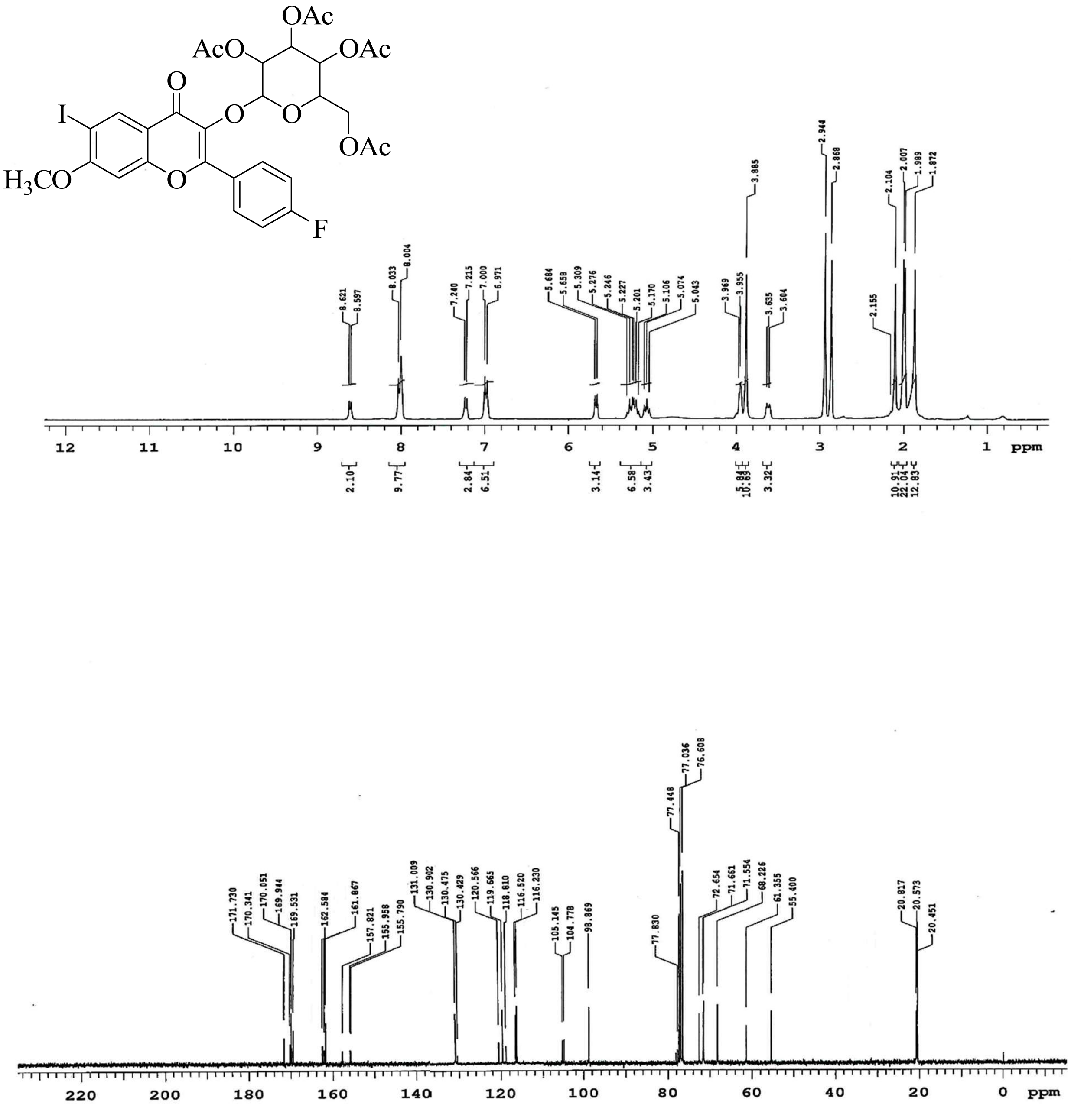

Figure S1.14: ^{1}H- and ^{13}C-NMR spectra of **2n** in $CDCl_3$ at 300 and 75 MHz, respectively.

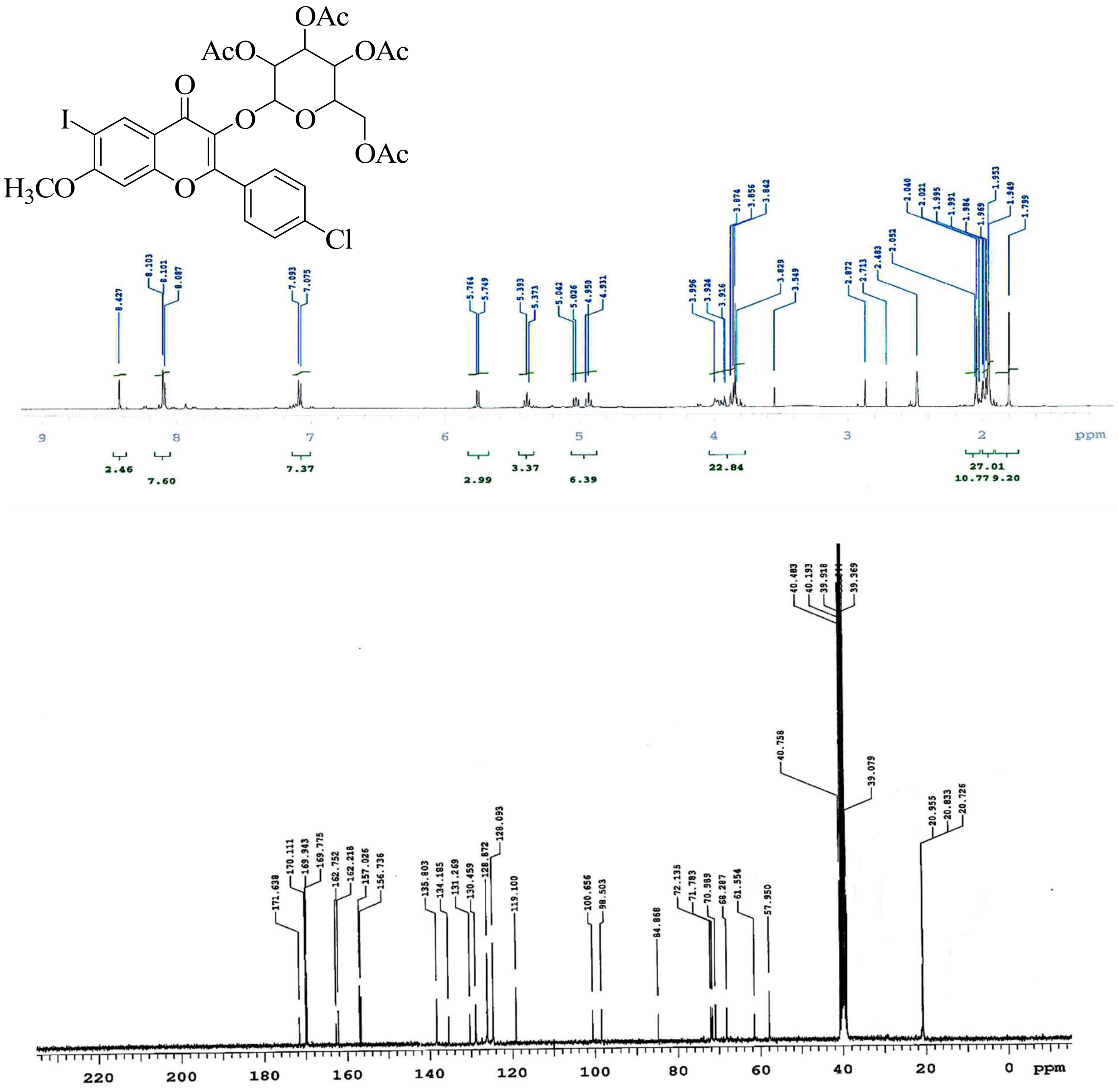

Figure S1.15: ^{1}H- and ^{13}C-NMR spectra of **2o** in DMSO-d_6 at 500 and 125 MHz, respectively.

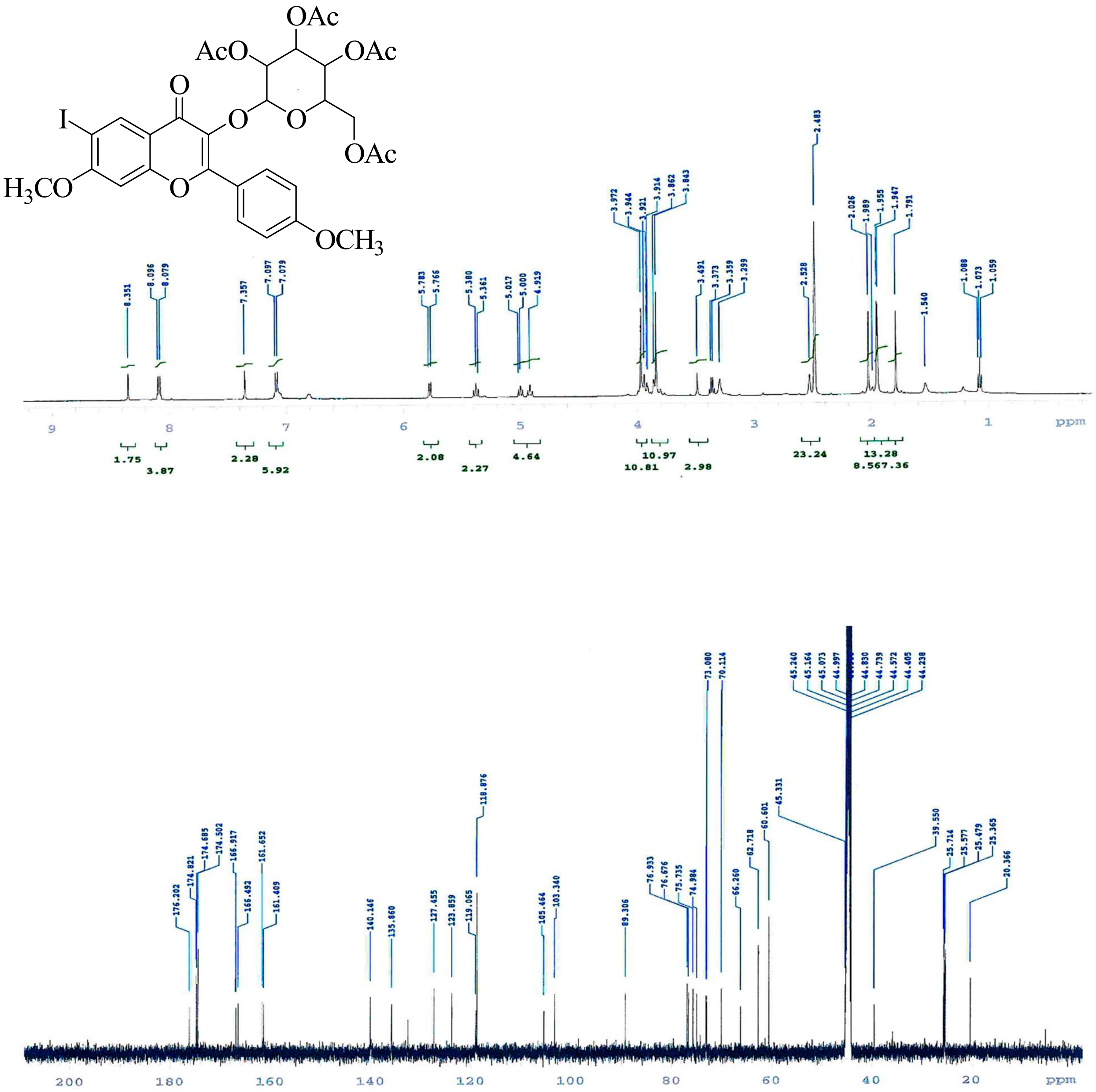

Figure S1.16: ^{1}H- and ^{13}C-NMR spectra of **2p** in DMSO-d_6 at 500 and 125 MHz, respectively.

Figure S2: **Lineweaver-Burk and Dixon plots for compounds 2l and 2p against AChE and BChE.**

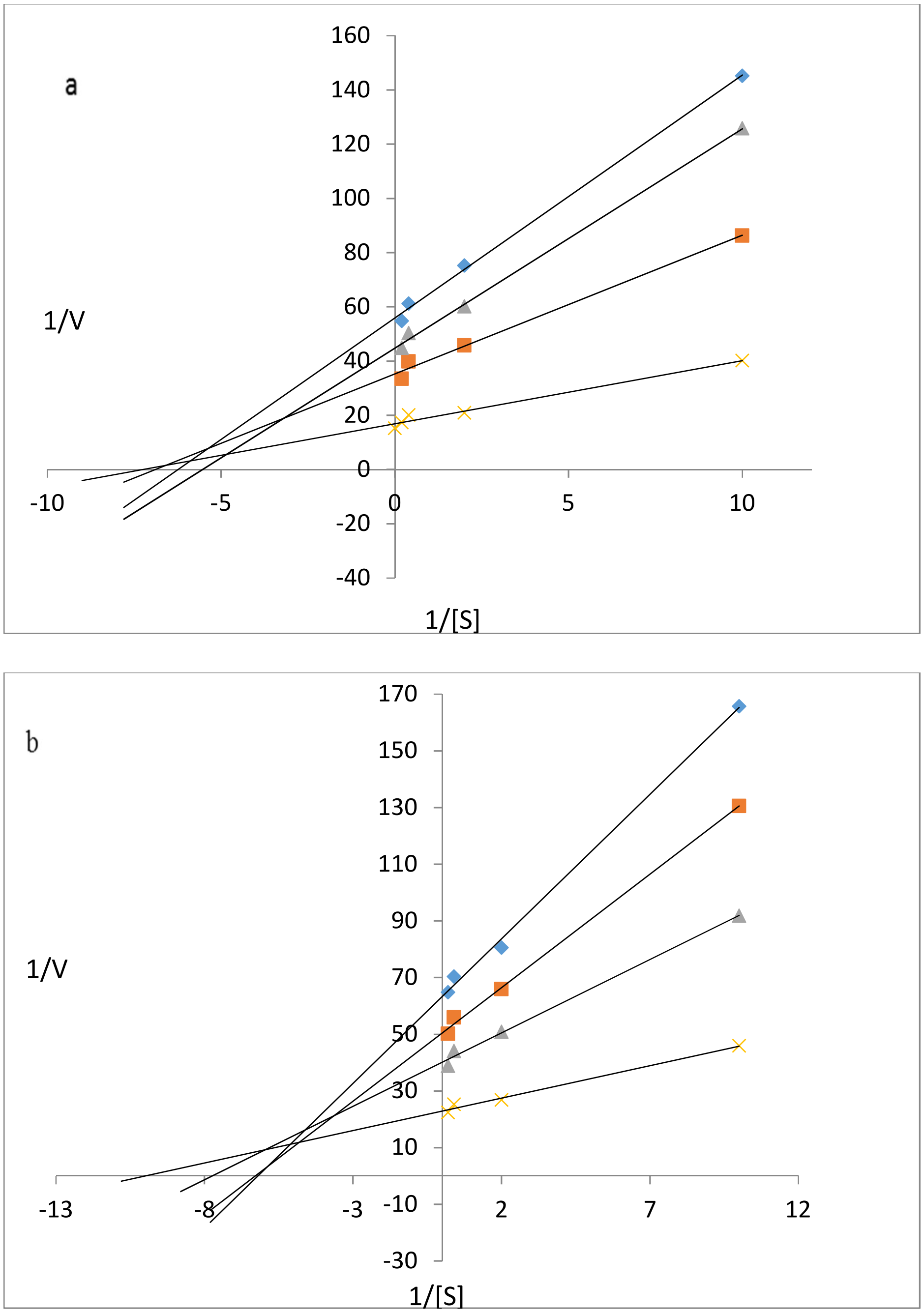

Figure S2.1: Lineweaver–Burk plots for inhibition of AChE by **2l** (a) and 2**p** (b). Yellow symbols and fitted straight lines represent enzyme activity in the absence of inhibitor, while grey (2.5 μM), orange (3.5 μM) and blue (5 μM) represent various concentrations of inhibitor.

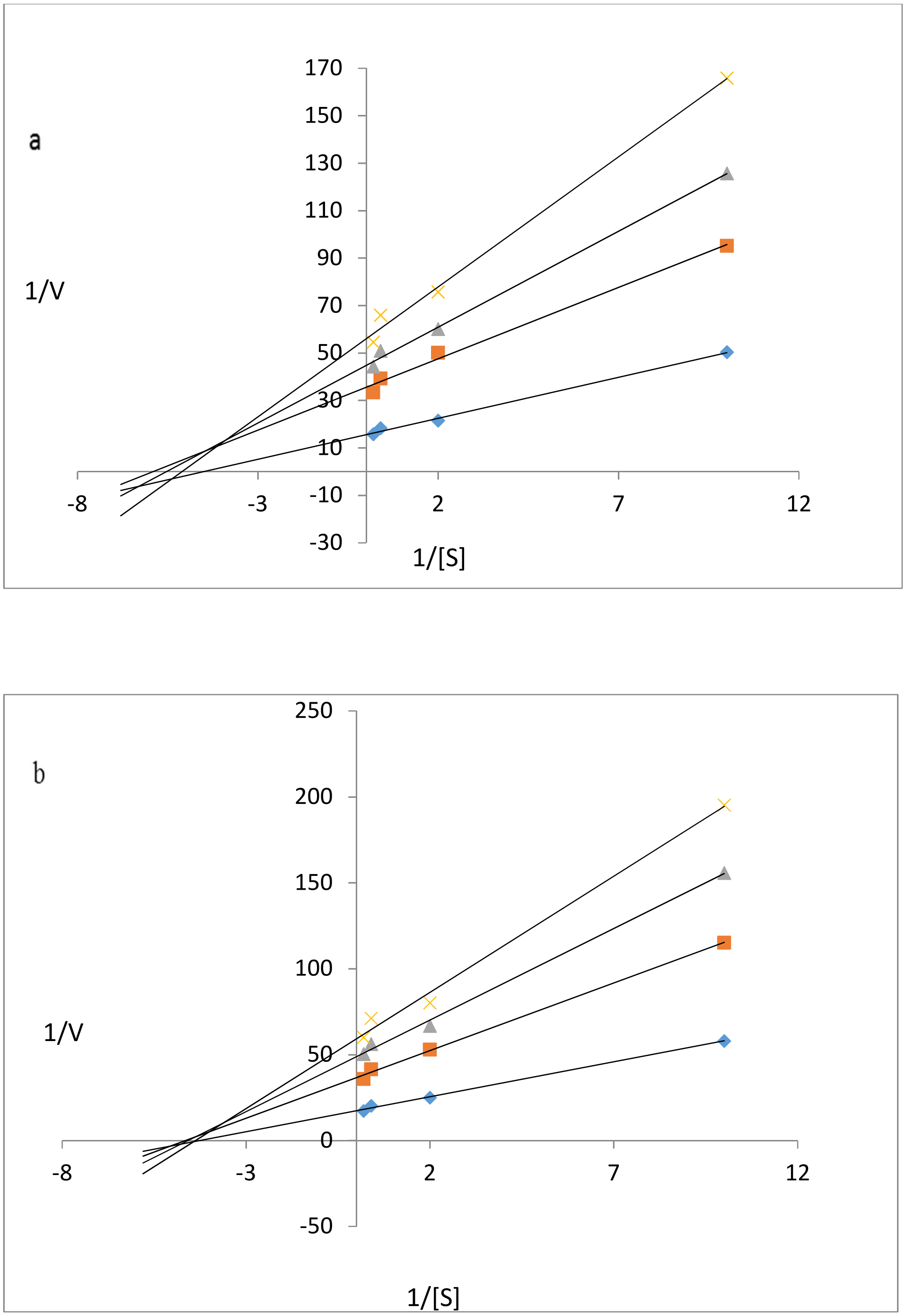

Figure S2.2: Lineweaver–Burk plots for inhibition of BChE by **2l** (a) and **2p** (b). Blue symbols and fitted straight lines represent enzyme activity in the absence of inhibitor, while orange (2.5 μM), grey (3.5 μM) and yellow (5 μM) represent various concentrations of inhibitor.

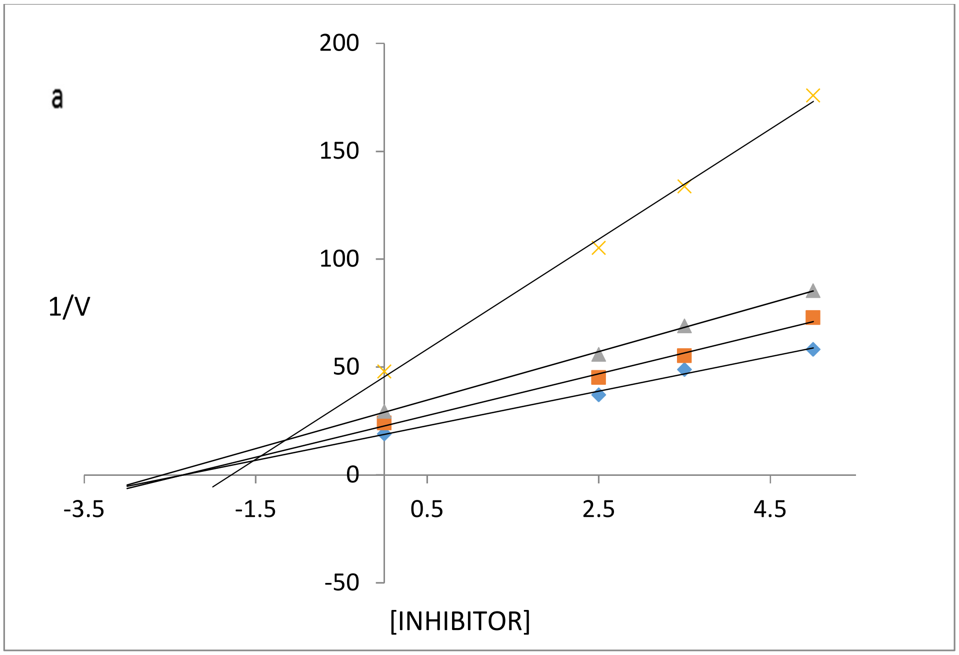

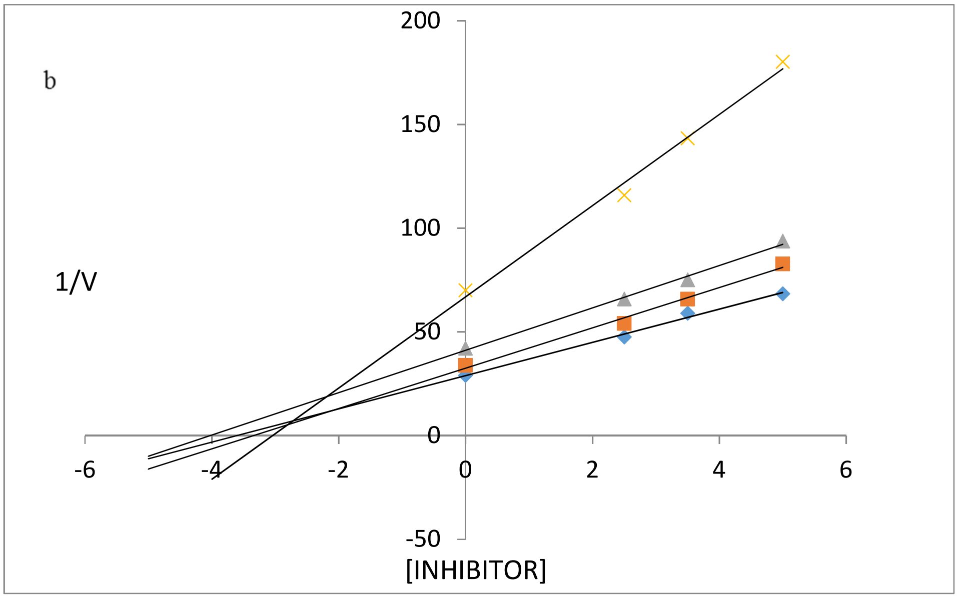

Figure S2.3: Dixon plots for inhibition of AChE by **2l** (a), **2p** (b). Blue symbols and fitted straight lines represent enzyme activity with 5 mM substrate, while orange (2.5 mM), grey (0.5 mM) and yellow (0.1 mM) represent various concentrations of substrate.

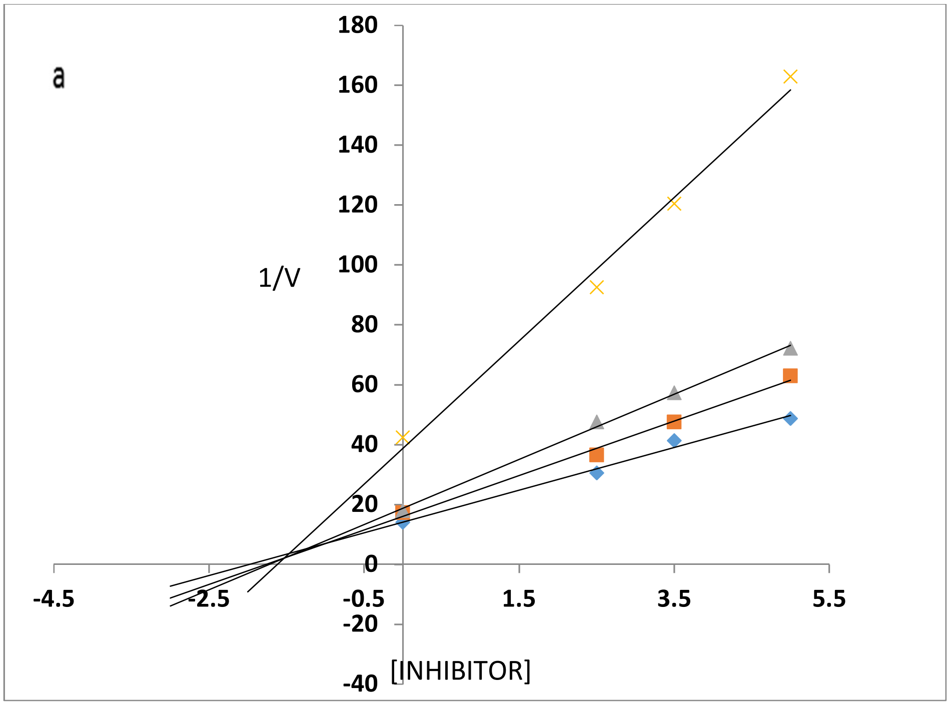

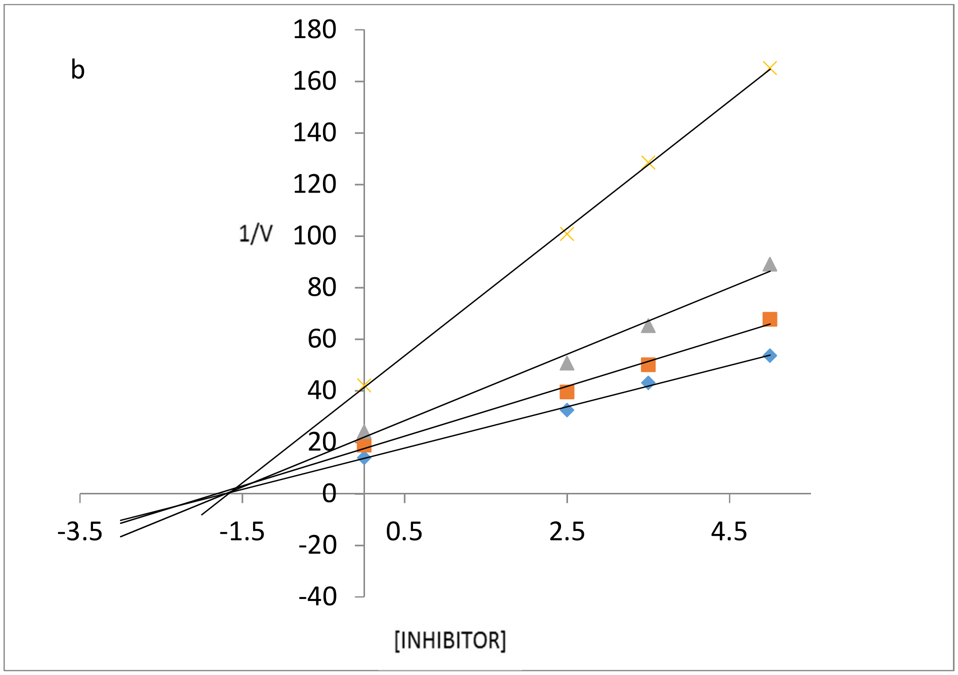

Figure S2.4: Dixon plots for inhibition of BChE by **2l** (a) and **2p** (b). Blue symbols and fitted straight lines represent enzyme activity with 5 mM substrate, while orange (2.5 mM), grey (0.5 mM) and yellow (0.1 mM) represent various concentrations of substrate.

Figure S3: **Lineweaver-Burk and Dixon plots for compounds 2l and 2p against β-secretase.**

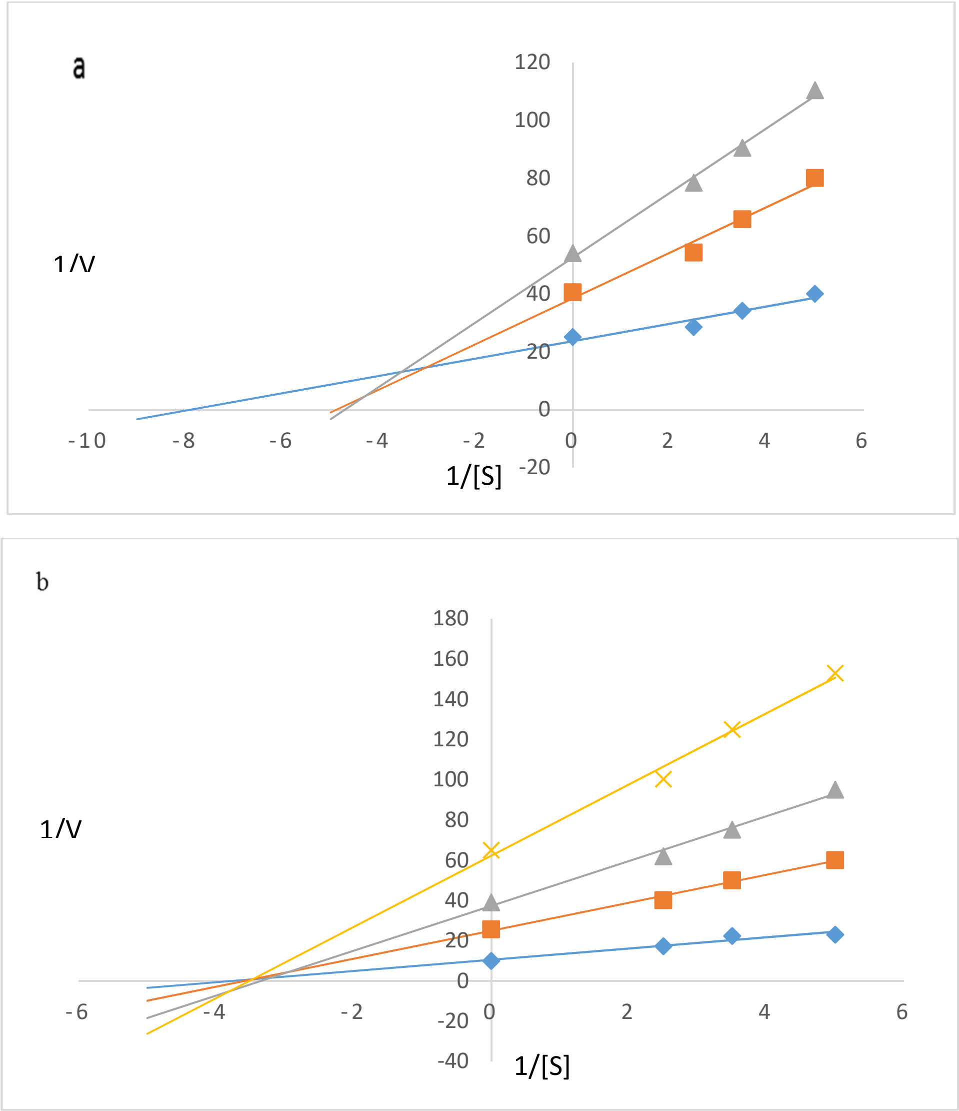

Figure S3.1: Lineweaver–Burk plots for inhibition of BACE-1 by **2l** (a) and **2p** (b). Blue symbols and fitted straight lines represent enzyme activity in the absence of inhibitor, while orange (2.5 μM), grey (3.5 μM) and yellow (5 μM) represent various concentrations of inhibitor

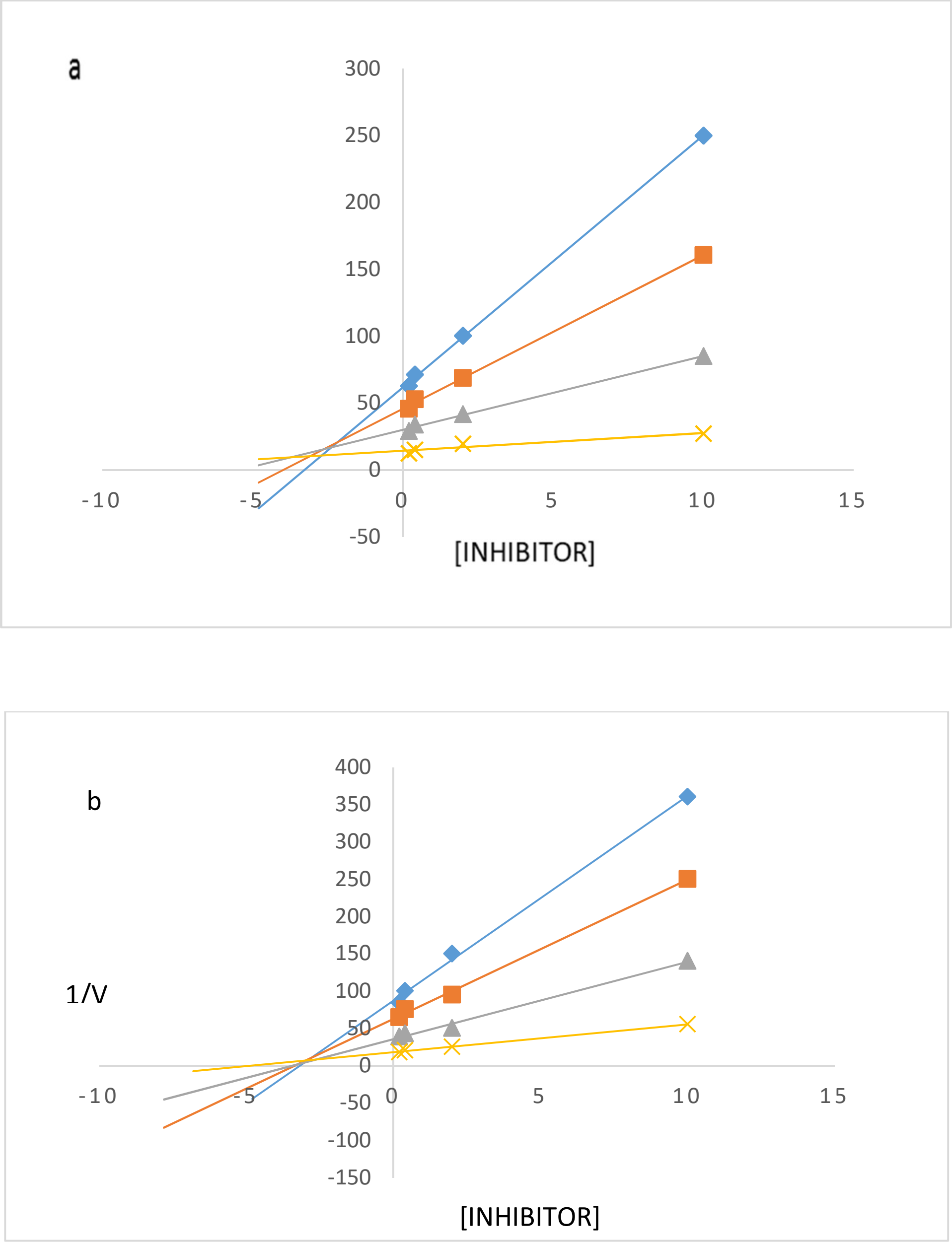

Figure S3.2: Dixon plots for inhibition of BACE-1 by **2l** (a) and **2p** (b). Blue symbols and fitted straight lines represent enzyme activity with 0.1 mM substrate, while orange (0.5 mM), grey (2.5 mM) and yellow (5 mM) represent various concentrations of substrate.
